# The Perfect Imperfections of Perovskite Oxide Catalysts in the Aspect of Defect Equilibria

**DOI:** 10.1002/smsc.202400386

**Published:** 2024-10-26

**Authors:** Maria Christy, Seunggun Choi, Jiseok Kwon, Jinwoo Jeong, Ungyu Paik, Taeseup Song

**Affiliations:** ^1^ Department of Energy Engineering Hanyang University 222 Wangsimni‐ro Seongdong‐gu Seoul 04763 Republic of Korea; ^2^ Department of Battery Engineering Hanyang University 222 Wangsimni‐ro Seongdong‐gu Seoul 04763 Republic of Korea

**Keywords:** defect engineering, electrocatalysis, perovskite oxides

## Abstract

ABX_3_ (X = O) perovskite oxides are an uprising class of alternative electrocatalysts in eminent technologies like electrocatalysis, photocatalysis, thermocatalysis, and energy storage. The perquisites of perovskite oxide catalysts encompass ordered atomic structure, structural/compositional extensibility, flexible electronic structure, lucrativeness, and so on. The ingenuity to precisely control and tune the inherent properties by reconstructing their crystal structure is particularly advantageous in electrocatalysis reactions like oxygen reduction and evolution reactions (ORR and OER). Incorporating multidimensional imperfections in the presumably perfect crystal structure of the perovskite catalysts is garnering booming attention among researchers. This concept can expertly influence the electronic structure and boost the reaction kinetics during electrocatalysis. Defects or imperfections are achieved by substituting A‐ and/or B‐sites with heteroatoms or by oxygen vacancies. Defect engineering points to a promising new direction in the development of perovskite oxide catalysts. This work surveys the recent progress in defect engineering and how it plays a vital role in their design, and application in electrocatalysis, mainly ORR/OER. The architecture, dimensionality, and the types of perovskite oxides based on their cations, crystal structures, and stoichiometries are surveyed for a comprehensive understanding. This review aims to provide an extensive outlook on oxide perovskite catalysts concerning structural defects.

## Graphical abstract

The ingenuity to precisely control the inherent properties by reconstructing the crystal structure is particularly advantageous in oxygen catalysis. Incorporating imperfections in the presumably perfect perovskite structure positively influences its electrocatalytic properties.

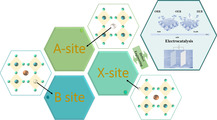

## 1 Introduction

**Figure 1:**
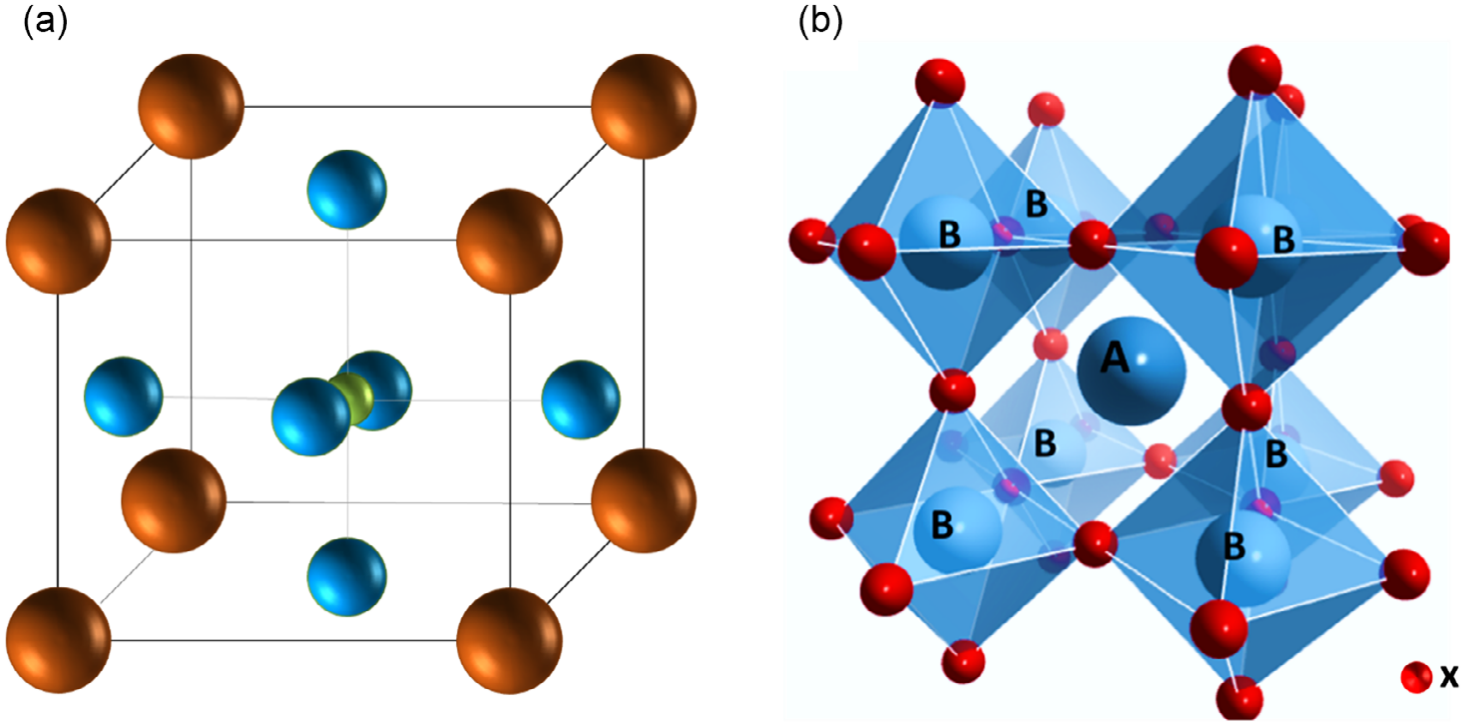
a) Perovskite CaTiO_3_ with a cubic unit cell showing Ti atom (brown), Ca atom (green), and oxygen atoms (blue). b) Crystal structure of a ABX_3_ perovskite structure.

Perovskite is a naturally occurring mineral first discovered in the Ural Mountains in 1839 and named in honor of a Russian mineralogist Lev Alekseyevich von Perovski (1792–1856) by another Russian mineralogist Gustav Rose (1798–1873).^[^ ^1^, ^2^ ^]^ This inorganic solid is typically composed of calcium, titanium, and oxygen in the form of CaTiO_3_ and has a cubic unit cell where Ti atoms occupy the corners (brown), Ca atoms (green) occupy the center, and oxygen atoms (blue) occupy at the midpoints of the edges (refer to **Figure** **1**a). The generic form of the perovskite is represented as ABX_3_, where A, B, and X are Ti, Ca, and O, respectively.^[^ ^3^, ^4^ ^]^ A perovskite structure is anything that has the same generic form (ABX_3_) and possesses a similar crystal structure as the original perovskite. The lattice arrangement of a perovskite structure can be described in detail as follows: a large atomic or molecular cation of type A is in the center of a cube usually with a +2 charge occupied by alkaline earth metals like Ca, Sr or rare‐earth elements like La, Y, and so on; a smaller cation of type B is at the corners of the cube commonly of +4 charge filled by transition metals; and a negatively charged anion is at the faces of the cubes commonly occupied by oxygen. Thus, a basic ideal cubic perovskite structure encompasses a larger A cation in a 12‐fold octahedral, and a smaller B cation with a sixfold cubic coordination and oxygen atoms that completes the unabridged structure (refer to Figure 1b).^[^ ^5^, ^6^, ^7^ ^]^ The valence state of the larger 12‐fold coordinated A‐site cation is 1^+^, 2^+^, or 3^+^ and that of the sixfold coordinated B‐site cation is usually 3^+^, 4^+^, 5^+^, or 6^+^.^[^ ^8^ ^]^ Regardless, by employing appropriate A, B, and X ions, more than 2346 single perovskite (SP) oxides can be constructed.^[^ ^9^ ^]^ Further, umpteen perovskite structures can be potentially created by full or partial substitution of A, B, and X sites, which will be surmised in a later section.^[^ ^10^ ^]^

The unique crystal structure and nonstoichiometric oxygen involving a wide range of ions make these frameworks surpassingly pliable for chemical tailoring.^[^ ^11^ ^]^ The broad range of occupants with variable composition and design bestows the structure with their expansive physicochemical features. Depending on the atoms or molecules used in the architecture, the extensive properties of the resulting perovskites range from superconductivity to giant magnetoresistance, optoelectronic properties, not to mention electrocatalytic characteristics, and many more.^[^ ^12^, ^13^ ^]^ Exploiting this designing extensibility, it is plausible to develop exclusive perovskite structures with befitting elements for any desired application.^[^ ^14^, ^15^, ^16^, ^17^ ^]^ Perovskites are broadly explored for diverse environmental and energy applications concerning a viable future.

Population and economic growth of the modern world have also exponentially increased the demand for clean and sustainable energy. Thus lately, a high volume of research is steered toward energy storage and conversion with extreme regard to environmental remediation. Renewable energy sources like electrochemical devices, namely, batteries and fuel cells, are replacing the traditional power sources and focusing on minimizing the carbon foot print. By harnessing the electrochemical route, safe, green, sustainable, and environment‐friendly energy sources can be achieved. The core electrochemical processes that take place in energy storage and conversion systems like metal–air batteries and water electrolyzer include oxygen reduction, oxygen evolution, and hydrogen evolution reactions (ORR, OER, and HER). These electrochemical processes employ electrocatalysts where their sluggish kinetics still prevent them from practical applications. As a result, polluting the ecosystem is unending and the need for developing advanced electrocatalysts is pressing. Perovskites have garnered comprehensive attention as electrocatalyst owing to their unique qualities. Since the first catalytic performance reported in the early 1970s, perovskites have shown tremendous growth with exceptional catalytic properties even suggesting the possibility of replacing the standard platinum‐group metals catalysts. Distinctively, the added merits like structural stability, thermal stability, oxygen properties (mobility, vacancy), electron mobility, electrical conductivity, redox property, tunable structural and electronic characteristics, and so on make them desirable for effective heterogeneous catalysis in versatile catalytic reactions (electrocatalysis, photocatalysis, thermocatalysis, and so on) and energy storage devices like batteries, fuel cells, and supercapacitors.^[^ ^18^, ^19^, ^20^ ^]^

It has been ascertained that the functional properties of a perovskite structure can be influenced by understanding and controlling their chemical defects. That is, by altering the crystal symmetry, tweaking the A/B occupants, or by creating oxygen vacancies, their intrinsic characteristics can be steered toward an optimized output. Crystal symmetry directly control the atomic distances which, in turn, could pilot the electron transport, site distances, and catalytic performances. Substituting appropriate A‐ and B‐site cations has been reported to substantially improve the bifunctionality of the catalyst material.^[^ ^21^, ^22^ ^]^ Further, oxygen‐deficient perovskite oxides are among the prevalently studied catalysts since their high oxygen ion mobility that can greatly manipulate the surface chemistry and overall catalytic property. Hence, defect engineering has accumulated a spotlight among researchers to probe the surface and interfacial properties of the perovskite structured catalyst materials.^[^ ^23^, ^24^, ^25^, ^26^, ^27^ ^]^

Herein, we attempt to review the types of perovskite oxide and their defect equilibrium, and principally delve into the defect engineering of perovskite structures for optimized catalytic application. There have been colossal advances in the successful structural engineering of perovskites with enhanced catalytic properties since the 1960s. It is necessary to understand the disparate areas of such progresses including theoretical, experimental, and simulation methods. This work surveys the critical role of defect engineering in the design, fabrication, and application of oxide perovskite catalysts. This detailed review will help the readers understand the importance of defect engineering, its impact on material characteristics, and the tremendous possibilities of perovskite catalysts in the future of catalysis.

## 2 Formation of Perovskites

To understand the architecture and dimensionality of perovskites, it is highly essential to apprehend the crystal structures.

### 2.1 Crystal Structure

**Figure 2:**
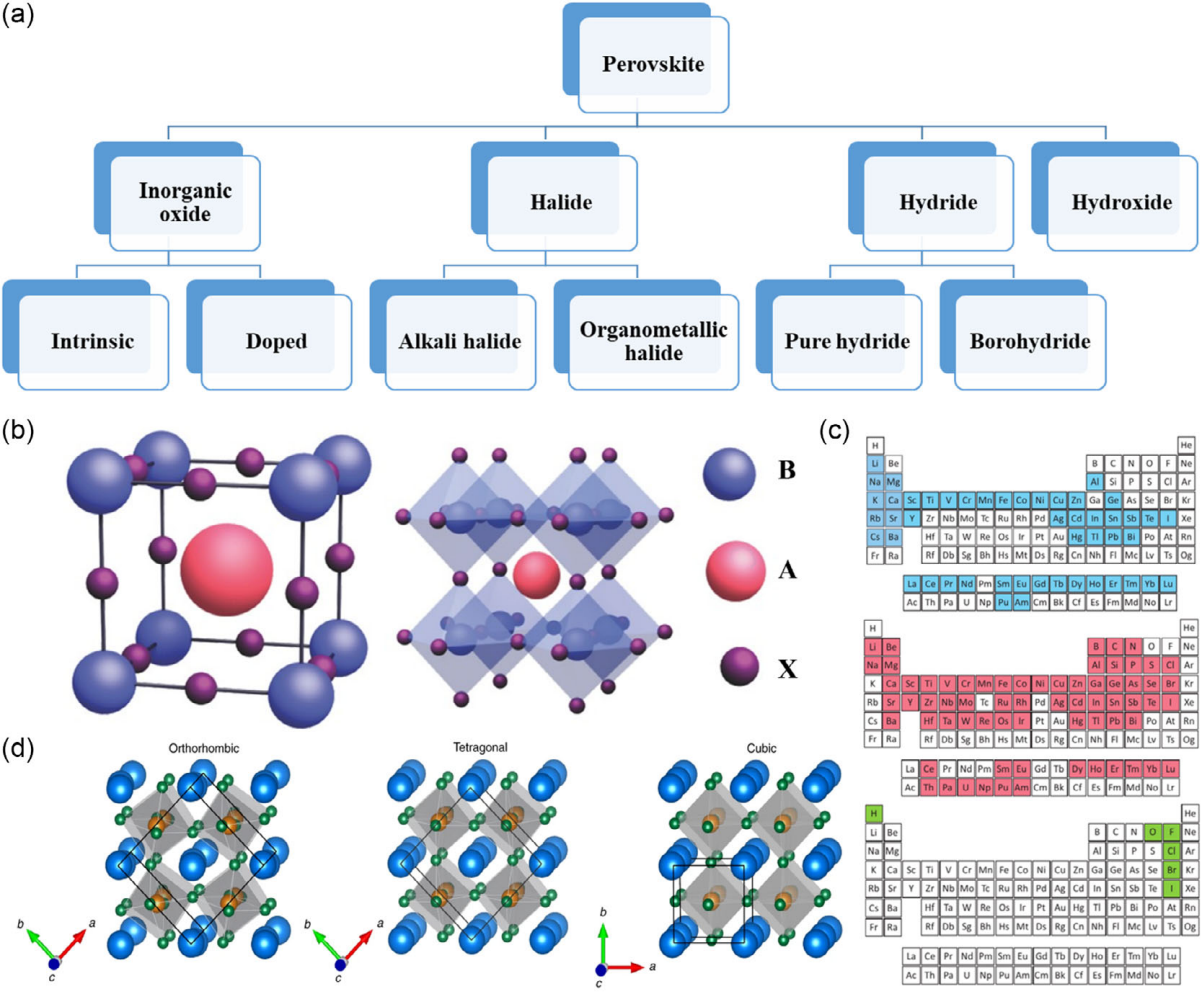
a) Classification of perovskite structure formation based on anion X. b) The perovskite ABX unit cell showing a large A and small B cations, and X anions including the octahedron formed by X anions and the octahedra lattice. Reproduced with permission.^[^ ^116^ ^]^ Copyright 2018, Advanced Science. c) Periodic table representing the possible A‐site cations (blue), B‐site cations (pink), and possible X‐site anions (green). Reproduced with permission.^[^ ^117^ ^]^ Copyright 2020, CRC Press. d) Perovskite lattice with different symmetries like orthorhombic and tetragonal along with cubic lattice structure. Reproduced with permission.^[^ ^118^ ^]^ Copyright 2021, Springer Nature.

As mentioned, the general formula for a SP structure is ABX_3_, with A and B cations forming the sublattice and body‐centered‐cubic structure, respectively. Most importantly, the formation of a perovskite structures depends on X anion. X is a −2 negative anion which is usually oxygen, or otherwise a halogen (F, Cl, Br, I). In general, the cation's valence permutations for oxide perovskites can extend to A_1_ + B_5_ + X_3_^2−^ and A_5_ + B_1_ + X_3_^2−^, while that of halide perovskites are confined to A_1_ + B_2_ + X_3_. Thus, depending on the X anion, perovskite can be named as 1) inorganic oxide perovskite (X = O), 2) halide perovskite (X = F, Cl, Br, and I), 3) hydride perovskite (X = H), and 4) perovskite hydroxides (X = OH). Hybrid structures are also possible, such as organic–inorganic mixed halide perovskite (X = hybrid). The possible formation of perovskite structures according to anion X is illustrated in **Figure** **2**a. The main structures can further be divided into subgroups, raising the total number of possible SP structures to be more than 3882.^[^ ^28^ ^]^ Perovskites can harbor most of the metals from the periodic table (≈90%) and a compelling amount of anions in their structure. All the potential cation and anion candidates that can occupy A, B, and X sites (Figure 2b) are shown in Figure 2c for a comprehensive understanding. The periodic tables exhibit the feasible A‐site cations (blue), B‐site cations (pink), and X‐site anions (green) to occupy the perovskite structured materials. While A‐site is paramount for the structural integrity, B‐site governs the intrinsic properties of the as‐formed perovskite structure.^[^ ^29^, ^30^ ^]^ Among these, oxide perovskites are predominantly investigated for catalytic applications and halide perovskites are widely recognized as one of the most promising materials in light‐emitting devices and photovoltaics.^[^ ^31^, ^32^, ^33^, ^34^, ^35^, ^36^ ^]^ More specifically, transition metal‐containing perovskite oxides are known to be endowed with intrinsic properties equivalent to that of noble metal catalysts. Other than pristine oxide perovskites, hybrid perovskites have been reported to achieve higher catalytic activity. Novel hybrids by composing a first Ruddlesden–Popper (RP) phase or other layered phases with a second perovskite phase have been reported as outstanding OER electrocatalysts.^[^ ^37^ ^]^

### 2.2 Lattice

The ideal crystal formation of a perovskite is perceived to be a high‐symmetric cubic Bravais lattice of space group Pm3m due to the orderly arranged octahedra.^[^ ^38^ ^]^ That is, a central A ion within a cubic B sublattice and the B cation is coordinated by six octahedral X ions (Figure 2b). This ideal condition might not be true in most cases where the structures do not possess a cubic symmetry, but are distorted based on the ionic radii, interatomic distance, and the stacking of A, B, and X elements. These factors commonly result in the formation of tetragonal, orthorhombic, or hexagonal symmetries (refer to Figure 2d). The disparity in the ionic radii results in the deformation of BO_6_ octahedra, which effectively promotes the metamorphosis with a considerable tilt in the lattice structure. According to Glazer et al. there are as many as 23 possible variants of lattice tilt possible that have been condensed to 14 according to their crystal symmetry.^[^ ^5^ ^]^ Nevertheless, so far, only four types of Glazer tilts have been experimentally realized, viz., Pnma, R3c, Pm3m, and I4/mcm, with 14 different space group symmetry.^[^ ^39^, ^40^ ^]^ Further, the stable crystal structure of a perovskite material is quantified using an empirical index of Goldschmidt tolerance factor (*t*). An ideal perovskite is expected to have *t* = 1, while in reality, the distorted perovskites exhibit values in the range of 0.7 ≤ *t* ≤ 1.0.^[^ ^41^ ^]^ For instance, when *t* < 0.71 or *t* = 0.71 ≈0.9, the octahedral framework is rearranged to form a (orthorhombic or rhombohedral) or (trigonal or orthorhombic or tetragonal) structures with lower symmetries, respectively. Even the most cubic perovskite exhibits a *t* value of 0.9. The Goldschmidt tolerance factor *t* is calculated using Equation (1):^[^ ^42^ ^]^

**Table:** 

	*t* value	Structure
(1) $t=\frac{R_{A}+R_{O}}{\sqrt{2}(R_{B}+R_{O})}$	>1	Hexagonal tetragonal
	0.9–1	Cubic
	<0.9 (0.71–0.9)	Rhombohedral orthorhombic

where *R*_A_, *R*_B_, and *R*_O_ are the ionic radii of the A and B cations, and X anion, respectively.

### 2.3 Nonstoichiometry

Electroneutrality is another factor besides ionic radii (*R*_A_, *R*_B_, and *R*_O_) that determines the structure of perovskite oxides. In ideal perovskites, the sum of charges of cations (A and B) equals the sum of charge of anion O which is generally attained by proper charge distribution among the elements. Yet, practically, deficiencies of cations (A, B) or anions (O) are more common that results in the formation of defective perovskites. Example for anion or oxygen deficient perovskites is the brownmillerite La_2_Ni_2_O_5_. Since cation nonstoichiometry is the opposite, oxygen excess can occur in this case. Examples for B‐site and A‐site vacant perovskites are hexagonal Ba_5_Ta_4_O_15_ and Cu_0.5_TaO_3_, respectively, whose detailed characteristics will be discussed in the later section.

## 3 Classification of Perovskites

### 3.1 Structure

Depending on the elements and their arrangement, perovskites are basically classified into 1) single, 2) double, 3) layered perovskites, and so on.

#### 3.1.1 SPs

SPs are basic perovskite structures with the original generic formula ABX_3_ (Figure 1 and 2a). They vary from a low symmetrical triclinic to a highly symmetric cubic‐phased structure. Oxygen‐deficient SP with transition metals at the B‐site and alkaline/rare‐earth metals at the A‐site are the most commonly investigated.^[^ ^43^, ^44^ ^]^ They are easy to synthesize at low temperatures and can be mindfully modified according to a purpose. Their architectural flexibility holds the feasibility to accommodate diverse elements with rich chemistry, thus enabling the fine‐tuning of their physical and chemical properties.^[^ ^45^ ^]^

#### 3.1.2 Double Perovskites

**Figure 3:**
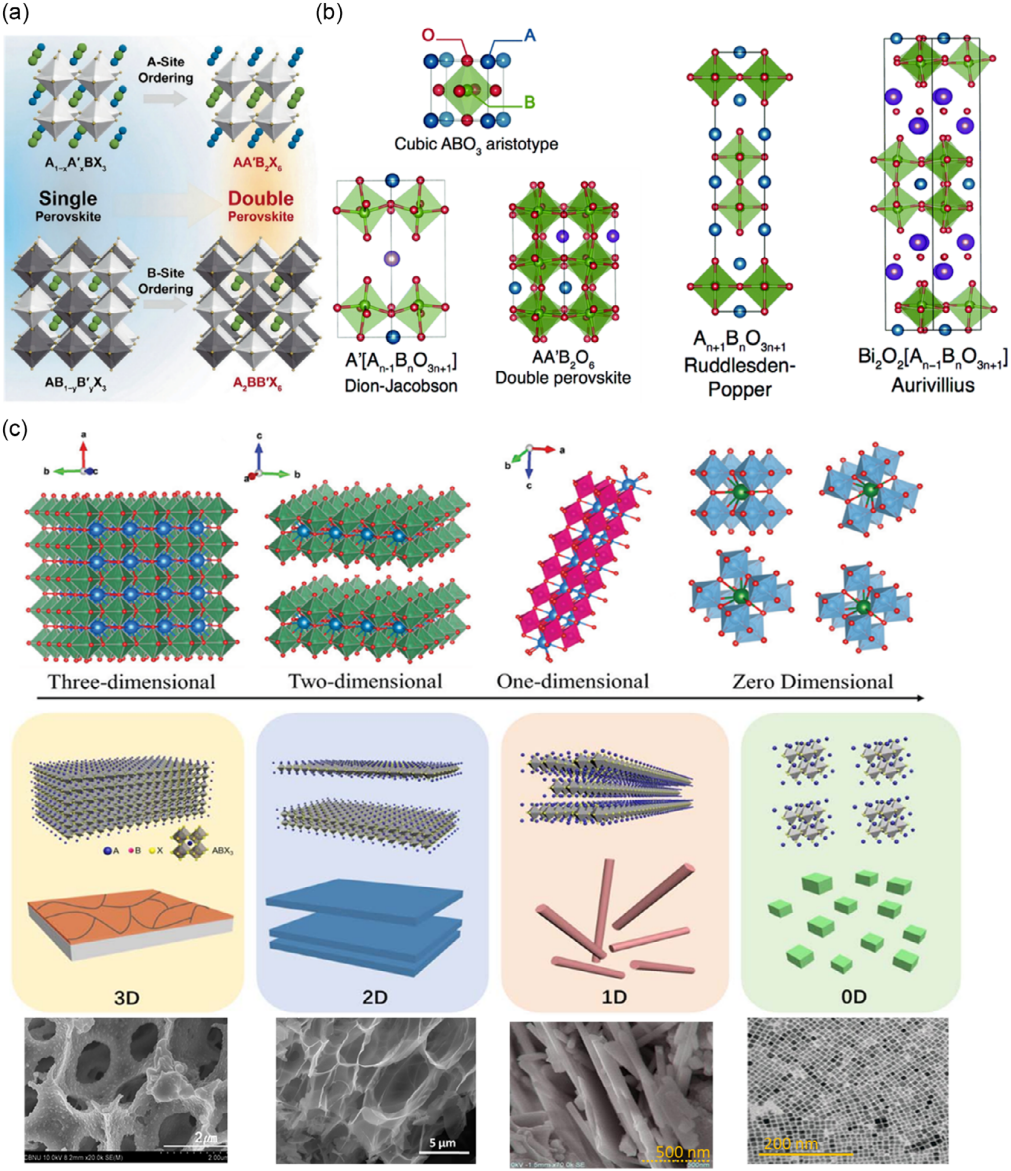
a) Formation of SP and DP structures. Reproduced with permission.^[^ ^119^ ^]^ Copyright 2023, Springer. b) Layered perovskites derived from the cubic ABO_3_ aristotype showing Dion–Jacobson phase, RP phase, and Aurivillius phases. Reproduced with permission.^[^ ^120^ ^]^ Copyright 2015, RSC. c) Dimensional perovskites in different dimensionalities (0, 1, 2, and 3 D) and their corresponding morphologies. Reproduced with permission.^[^ ^19^, ^72^, ^121^, ^122^, ^123^, ^124^ ^]^ Copyright 2021, RSC; 2020, MDPI; 2017, Wiley; 2016, RSC; 2017, Elsevier; and 2013, Wiley, respectively.

Double perovskites are structures with the formulae A_2_BX_6_ (A′A″BX_6_) or AB_2_X_6_ (AB′B″X_6_) where a partial cation substitution at A or B or both can be achieved.^[^ ^46^ ^]^ Double perovskites (DPs) are garnering increasing interest since they can host multiple 3*d* metal cations at the A–B sites that would expand the interaction between the localized 3*d* transition metals and the relatively delocalized 4*d*/5*d* transition metals within the same frame. Two types of BO_6_ octahedra can double the lattice spacing. Also, DP has been reported to outperform SP with double B‐site cations or two types of property‐determining cations in optoelectronic as well as electrocatalytic applications. The structures are further extended to A_2_A′B_2_B′O_9_ triple‐cation or AA′_3_B_4_O_12_ quadruple‐cation perovskite structures, and so on (A = Sr, Ba, La, and so on; B, B′ = transition metals like Mg, Zn, Nb, Ta, and so on).^[^ ^47^ ^]^ A DP could be easily perceived as a cubic perovskite structure generated by stacking two SP blocks, i.e., one of the cubic axes and the *c*‐axis doubled (**Figure** **3**a). Likewise, it is a triple perovskite when the unit cell is tripled or the *c*‐axis is triple as that of a SP structure, and so on.

#### 3.1.3 Layered Perovskites

Multiple perovskite layers are assembled to form a layered structure commonly called as layered perovskites. Layered perovskites can be divided into Dion–Jacobson phase, RP, and Aurivillus phase (Figure 3b).^[^ ^48^, ^49^ ^]^ The Dion–Jacobson phase, A′[A_*n*−1_B_*n*_O_3*n*+1_], has one interlayer cation per formula unit and RP phase, *A*2′[A_*n*−1_B_*n*_O_3*n*+1_], has two interlayer cations per formula unit. In case of Aurivillus phase, Bi_2_O_2_[A_*n*−1_B_*n*_O_3*n*+1_], a covalent network of (Bi_2_O_2_)^2+^ in the middle holds the 2D perovskite slabs. The term layered–rock‐salt intergrown structure can be used to understand better. There is still look‐out for new members of these layered perovskites because of their exceptional properties such as ion‐exchange, intercalation, catalysis, superconductivity, thermal stability, and so on. The choice of A‐ and B‐site cations, and the oxygen‐enriched layers of the layered structure render the above super properties.

#### 3.1.4 Hexagonal Perovskites

As the name says, perovskite structures that possesses a hexagonal closed packing of AX_3_ layers are called as hexagonal perovskites.^[^ ^50^ ^]^ Hexagonal perovskite oxides allow the face‐sharing of metal–oxygen octahedra in their frameworks, resulting in dimers, trimers, tetramers, or longer chains of face‐sharing octahedra in the crystal structures. Unlike cubic perovskites, the tolerance factor for hexagonal perovskite structures can be *t* > 1, displaying a large difference between *R*_a_ and *R*_b_. The close packing of hexagonal AO_3_ layers is stacked in cubic or hexagonal fashion by sharing their vertices and faces, respectively. Thus stacking, a number of hexagonal perovskite structures can be formed. A few examples include mixed ionic conducting hexagonal perovskites like Ba_6_R_2_Ti_4_O_17_ and Ba_7_Y_2_Mn_3_Ti_2_O_20_. Ba_6_R_2_Ti_4_O_17_ is formed with a 12H hexagonal polytype structure (space group *P*6_3_/*mmc*), composed by stacking the cubic and hexagonal BaO_3_ layers alternatively. In 2013, Grimaud et al. reported hexagonal perovskite oxides (Ba_6_Mn_5_O_16_ and Sr_6_Co_5_O_15_) with the electronic structure of transition metal oxides, face‐shared (f‐s) and prism (P) forming the hexagonal structure.^[^ ^51^ ^]^

#### 3.1.5 Anion‐Deficient or Cation‐Deficient Perovskites

Anion‐deficient phase or brownmillerite‐type perovskite structure is composed of alternative B0_6_ octahedra and B0_4_ tetrahedral layers.^[^ ^52^ ^]^ This is a type of ion vacancy ordering in oxygen‐deficient perovskite structures. These structures can accommodate one‐sixth of oxygen vacancies ordered in the alternating layers and can be described with a formula of A_2_B_2_O_5_ or A_2_BB′O_5_. Anion‐deficient structures mainly attracted attention due to their potential to incorporate additional oxygen atoms in their lattice, which exhibit high (ionic–electronic mixed) conductivity and fast oxygen ion conductivity.

ABO_3_‐type perovskite structure holds a ratio of one for A‐ to B‐site atoms. A‐site cation forms a 12‐fold coordination with oxygen ions via strong ionic bonds and B‐site cation coordinates with six oxygen via strong covalent bonds. Nonetheless, cation deficiency can be admitted in this structure up to 27% without eliminating the perovskite lattice. Cation or A‐site or B‐site deficiency is frequently used to modify the surface chemistry of perovskites and generate oxygen vacancies, mainly for catalytic applications.^[^ ^53^, ^54^ ^]^ Defect equilibria in perovskite oxides are generally expressed by Kröger–Vink notation which will be discussed in later sections.^[^ ^55^, ^56^ ^]^

#### 3.1.6 Hybrid Perovskites

Hybrid perovskites are prepared by linking two different perovskite structures with intriguing characteristics in order to corroborate the physicochemical characteristics in the product. Thus, prepared hybrid perovskites are found to enhance the oxygen ion diffusion during catalytic activity. Sun et al. developed hybrids formed by SP and DP structures which resulted in synergistic electrocatalytic HER performance, which was much better than any of the parent structures.^[^ ^57^ ^]^ Lately, 2D layered RP materials sandwiched between two rock‐salt layers, popular in the energy applications due to their multilayered structure, and higher surface‐to‐bulk ratios.^[^ ^58^, ^59^ ^]^ Hybrid perovskites are also formed with nonperovskite materials, for instance, interface engineering of RP perovskite with CeO_2_ and carbon heterojunction has been recently reported with interfacial coupling.^[^ ^60^ ^]^ The heterojunction has resulted in abundant new active sites by regulating the interface electron redistribution causing a high activity in the hybridized composite catalyst compared to the single counterparts.

### 3.2 Dimension

Alongside crystal structure, dimensionality also plays a crucial role in the electronic and catalytic properties of perovskite oxides. Perovskite structures can be explained with respect to the dimensions (0, 1, 2, or 3 dimensions) as follows (Figure 3). La‐based perovskite oxides stand out to be the most investigated structures for electrocatalytic application.

#### 3.2.1 0D Perovskite Structure

Basically, 0D materials (quantum dot, *x*, *y*, *z* < 100 nm) can have a physical dimension of several millimeters or larger, similar to bulk quantum materials.^[^ ^61^ ^]^ The size of the crystallites can directly influence their electronic and optoelectronic characteristics. 0D perovskites can be prepared by melting precursors, low‐temperature solution processes, room‐temperature self‐assembly, and so on. One of the best examples is 0D Cs_4_PbBr_6_ inorganic bulk perovskite structure.^[^ ^62^ ^]^ Perovskite quantum dots are predominantly applied for sensors, laser technology, cell imaging, quantum computing, and so on and most importantly in light emitting diodes, photocatalysis, and solar cells (photovoltaic).

#### 3.2.2 1D Perovskite Structure

1D (nanowire, *x* > 100 nm) perovskite structures are revolutionizing the modern‐day optoelectronic devices.^[^ ^63^ ^]^ The 1D morphology facilitates easy transport of charge and photons with low binding energies and long diffusion lengths. They also exhibit great compositional affability, tunable bandgap, and excellent stability at a molecular level. Perovskite nanowires can be prepared by controlled synthesis like self‐assembly or by direct methods like sol–gel and electrospinning, for example, (La_1−*x*_Sr_*x*_)(Mn_0.9_Ni_0.1_)O_3_ nanofibers prepared for supercapacitors and other energy applications.^[^ ^64^ ^]^ Liu et al.^[^ ^65^ ^]^ prepared perovskite La_1−*x*_Sr_*x*_CoO_3−*δ*_ (LSC) nanotubes by electrospinning technique and obtained exceptional bifunctional (ORR and OER) characteristics, owing to the stable hierarchical mesoporous/macroporous nanotubular structure.

#### 3.2.3 2D Layered Perovskite Structure

2D layered perovskite structures are based on [A_*n*1_B_*n*_O_3*n*+1_]_2_ layers, where *n* is the number of layers with interlayer metal cations (M_2_O_2_). These state‐of‐the‐art 2D nanosheets (*x*, *y* > 100 nm) are classified into RP, Dion–Jacobson, and Aurivillius phases, which have been mentioned earlier under the layered perovskite structures.^[^ ^48^, ^66^ ^]^

The general formula for RP phases can be described as M_2_[A_*n*1_B_*n*_O_3*n*+1_], where M and A are alkali, alkaline earth, or rare‐earth metals, M_2_ ions are interlayer cations, and B is a transition metal cation (Figure 3b). The interlayer gaps are highly likely to promote charge intercalation, which are of interest for application in Li‐ and Na‐ion battery anodes. One example can be the layer‐by‐layer assembly of 2D RP nanosheets with alternating C_8_H_17_NH_3_‐capped CsPb_2_Br_7_ nanosheets, i.e., layered (C_8_H_17_NH_3_)_2_CsPb_2_Br_7_ super lattice nanocrystals by Liu et al.^[^ ^67^ ^]^

The general formula for Dion–Jacobson phase is expressed as M[A_*n*−1_BnO_3*n*+1_], where M and A are alkali or alkaline earth metals, M is an interlayer cation, and B is a transition metal (Figure 3b). Dion–Jacobson phases have only one part (1 m) of the interlayers compared to RP. First, M[Ca_2_Nb_3_O_10_], where M is Li, Na, K, Rb, Cs, NH_4_, or Ti, was synthesized by Dion et al. followed by K[Ca_2_N_b_3O_10_] to H[Ca_2_Nb_3_O_10_] by Jacobson et al. with variable thickness.^[^ ^68^, ^69^ ^]^ The resultant low interlayer charge density is named as Dion–Jacobson phases which can be easily exfoliated to form monolayer colloids. These structures typically consist of triple perovskites interlinked with M sheets. The compositional flexibility of this phase can be exploited by employing thin‐film techniques allowing the synthesis of multiple layered perovskite structures. With controlled thickness, these ultrathin ferroelectric 2D perovskites are a boon for energy storage applications.

The Aurivillius phases have the general formula [Bi_2_O_2_]^2+^ [A_*n*−1_B_*n*_O_3*n*+1_]_2_, where A is an alkali or alkaline earth metal and B is a transition metal (Figure 3b). These are highly anisotropic 2D structures that interlink the Bi_2_O_2_ layers with BO_6_ layers of the perovskite structure by a weak van der Waals bond. One such example, Bi_2_MoO_6_, is a promising Aurivillius phase that possesses high electron and ionic conductivity applicable for catalysis and energy storage applications.^[^ ^70^ ^]^

All the above 2D layered perovskites are prepared by top‐down exfoliation or bottom‐up assembling strategies. Regardless of synthesis methods, the layered structures possess very high specific surface areas, high concentrations of active sites, and great stability that are widely attractive for energy and environmental applications.

#### 3.2.4 3D Perovskite Structure

In the perovskite lattice structure, the infinite corner‐sharing of the BX_6_ octahedra creates a compulsive repetitive architecture that forms a 3D structure.^[^ ^8^, ^71^ ^]^ This infinite corner‐sharing network results in the formation of direct bandgaps with semiconducting properties which influences the material's electronic and catalytic properties. The unique compositional and structural flexibility of 3D perovskite oxides facilitates the development of structural design for specific applications. The typical crystal lattice of the 3D perovskite structure with a presumed cubic structure has a tolerance factor of 1 (≈0.9). When the tolerance factor is outside 0.8 ≤ *t* ≤ 1, they form lower dimensionalities (2D, 1D, and so on). 3D perovskite materials can be bulk, spherical, hollow, and so on with highly porous surface. They possess a large contact area and provide surplus active sites for catalytic reactions. 3D perovskite structures are comparatively easy to synthesize by conventional solid‐state method. Oh et al.^[^ ^72^ ^]^ prepared 3D ordered mesoporous LSC using poly(methyl methacrylate) templates and reported enhanced catalytic performance with the abundant active sites and a large specific surface area.

## 4 Defects in Perovskite Oxides

**Figure 4:**
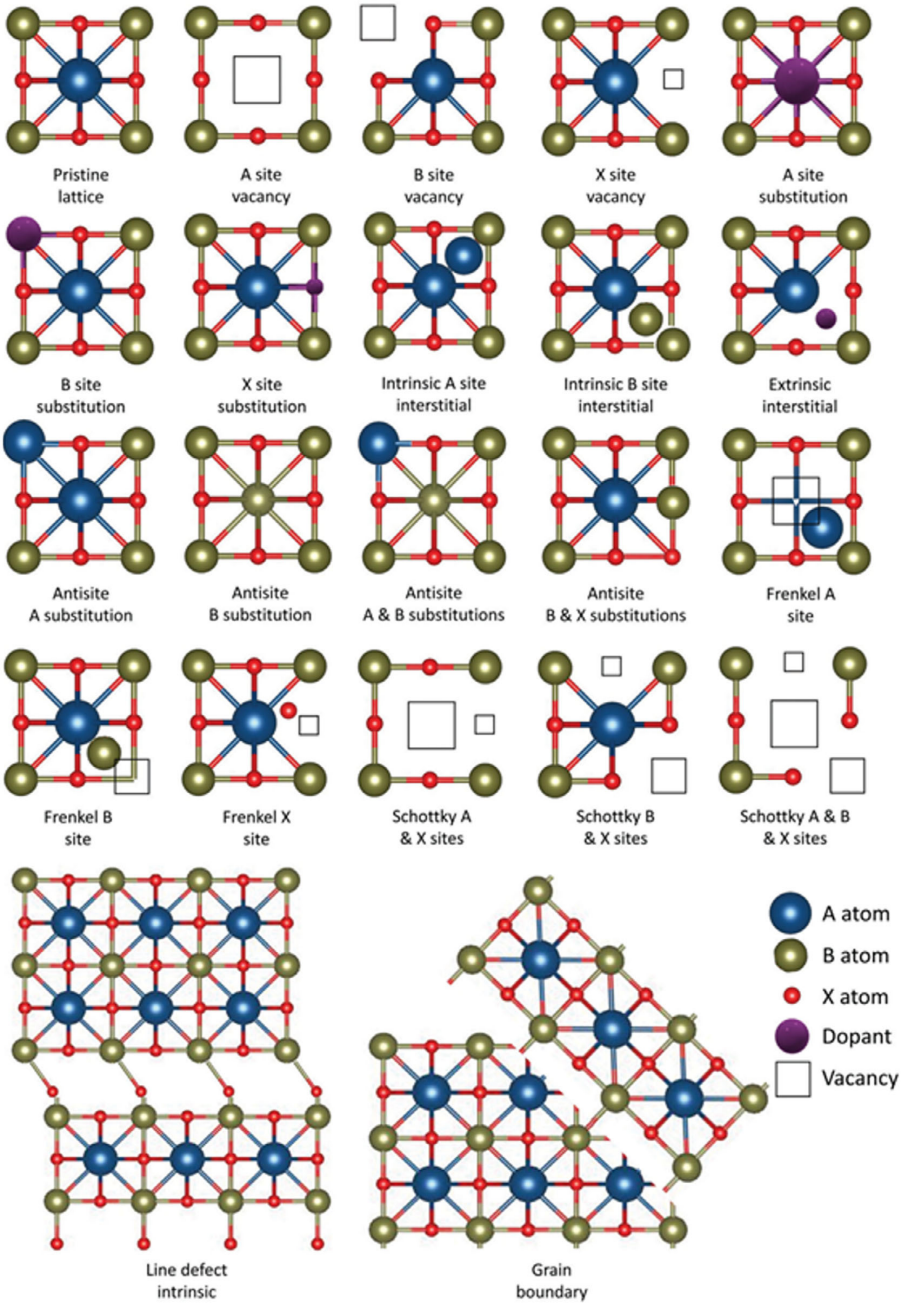
Types of intrinsic and extrinsic defects in perovskites. Reproduced with permission.^[^ ^19^ ^]^ Copy right 2021, RSC.

Defects in perovskite oxide can be basically divided into two main categories: point defects and extended defects. While point defects are localized defects that occur at a single lattice site of the crystal structure, extended defects occur over a larger area of the overall crystal structure. To fathom, point defects can be due to vacancy (atom missing) or substitution (atom replaced), and sometimes interstitial defects (atom occupying an interlattice site or an antisite). On the contrary, extended defects can caused by dislocations and grain boundaries. Dislocations are plainly lattice distortion, while grain boundaries are grains with different crystal arrays. Refer to **Figure** **4** for a comprehensive understanding on the different types of point and extended defects in the crystal lattice.

**Figure 5:**
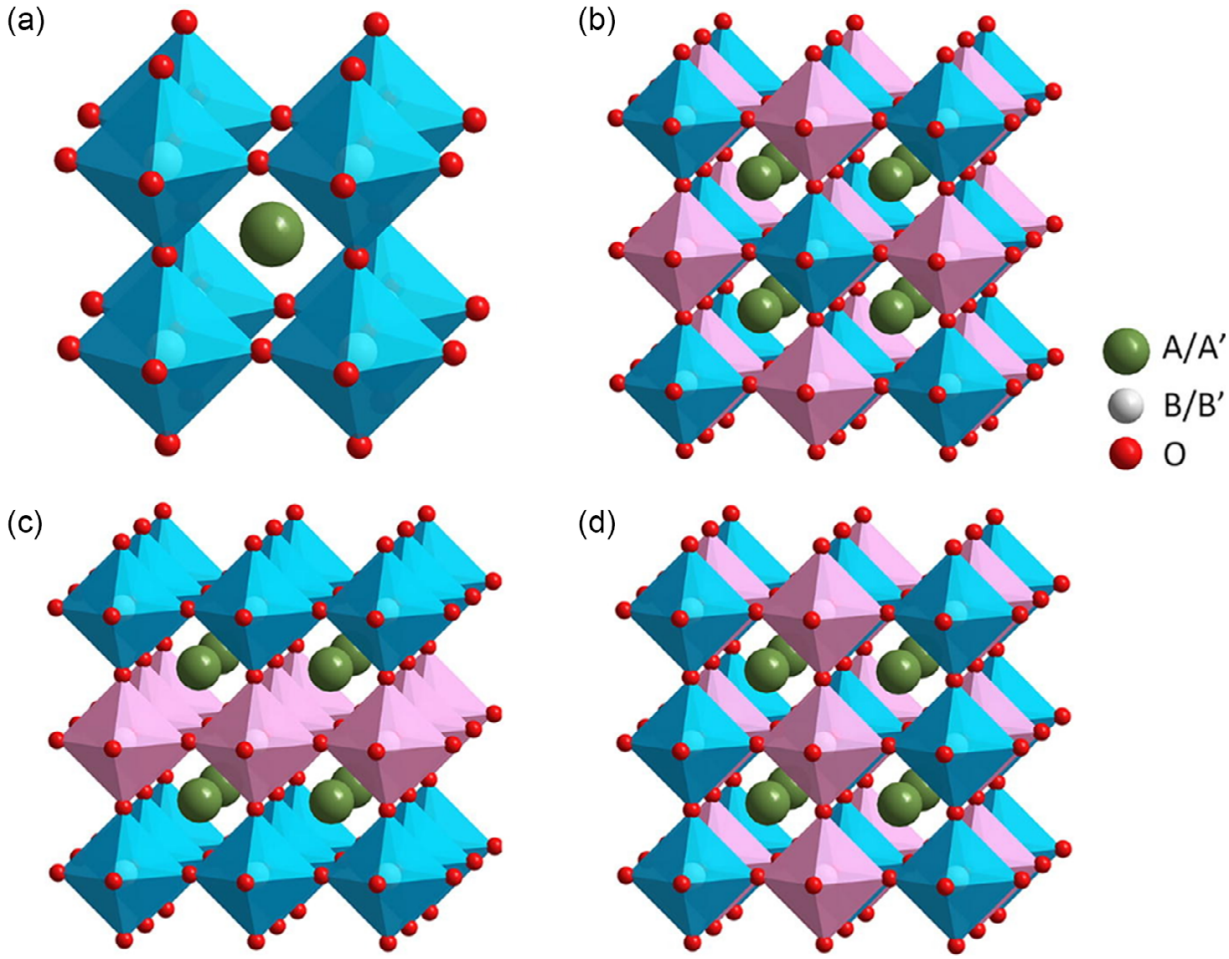
a–d) ABO_3_ perovskite structure, rock salt type structure, layered structure, and columnar structure, respectively. Conceptual summary and figure (a–d). Reproduced with permission.^[^ ^19^, ^125^ ^]^ Copyright 2021, Elsevier.

These lattice defects can be grouped as intrinsic or extrinsic based on their location such as defects that are within their own species or defects that are involved with an external species, respectively. Intrinsic defects can be caused by the disarray of atoms in the crystal lattice, for instance, point defects, lattice distortion, and so on are intrinsic.^[^ ^73^ ^]^ Extrinsic defects are created by impurity atoms like dopants (isovalent or aliovalent).^[^ ^74^ ^]^ Figure 4 also differentiates the types of intrinsic and extrinsic defects. In a typical ABO_3_ perovskite structure as in Figure 1b, the BO_6_ octahedron shares corners to form a 3D architecture. This structure where B‐site cations are occupied by transition metal ions with sixfold oxygen coordination is the simplest possible or a primitive ordering in an ordered perovskite oxide (**Figure** **5**a). When there is extrinsic defect like doping cations at A/B‐site, the lattice arrangement alters to accommodate the dopant. In that case, perovskite structures form interesting patterns like random, rock salt, layered, columnar, and so on.^[^ ^75^, ^76^ ^]^ It is common in most cases for the B and B′ cations to alternate in a 3D pattern to form a rock‐salt ordered‐type perovskite structure (Figure 5b). In some cases, when the B and B′ cations are connected in two different directions, they form a layered structure as in Figure 5c. Very rarely, when the B and B′ cations are connected in the same direction, columnar ordering take place as seen in Figure 5d which mainly occurs in DP structures like AA′BB′O6. The differently ordered perovskite patterns can be quantified by calculating the valence difference (Δ*V*) between B and B′ cations. Depending on whether Δ*V* ≤ 3, or 3 < Δ*V* ≤ 6, or Δ*V* = 2, the patterns can be random, or rock salt, or layered, respectively. Ordered perovskites or perovskite oxides with extrinsic defects are garnering interest for their superior catalytic performance owing to their oxygen defect concentration and the oxygen transport ability. Moreover, based on the occupation of metal ions in the A‐ and/or B‐sites, these perovskite oxides can act as oxygen conductors, proton conductors, mixed ionic–electronic conductors, and so on that are efficient for multifunctional catalysts.

The types of defects can be expressed using the Krögetr–Vink notation.^[^ ^73^, ^77^ ^]^ The common descriptors used in the Kröger–Vink notation are MM (meal ion at a metal sublattice site (M)), OO (oxygen at an oxygen sublattice site (O)), M’_M_ (metal ion substituting in the metal sublattice (M) also known as acceptor dopant), M_i_ (metal ion interstitial (i)), M_S_ (substituted metal ion that has diffused to the surface (S)), V’_M_ (metal (M) vacancy), V_O_ (oxygen (O) vacancy) with no net charge, e’ (electron) negative charge, and h (hole) positive charge.^[^ ^77^ ^]^

### 4.1 Point Defects

#### 4.1.1 Cation Defects

A‐ and B‐sites constitutes the cations in perovskite oxide structures. Theoretical and experimental studies report favorable merits produced by the oxygen vacancies that are created by A‐site defects. The metal at A‐sites usually has a stable valence (e.g., La^3+^ and Sr^2+^). Their structure and stability showcase A‐site perovskite oxides as promising candidates for electrocatalysis. When A‐site is substituted or displaced, or even when A‐site is entirely unoccupied, it results in a new class of BX_3_ architecture, where the valence of B‐site ranges from 3/4^+^ to 6^+^. There are many kinds of A‐site perovskite crystal structures and spinel oxides.^[^ ^41^, ^78^ ^]^ These structures can directly influence the catalytic performance of the A‐site perovskite oxides. Their morphology with respect to the synthetic method can also affect the intrinsic characteristics.

Similarly, B‐site defects can affect the structural properties and more deeply the electronic characteristics of the material like bandgaps, charge mobility, and so on.^[^ ^79^, ^80^ ^]^ The B‐site cation defects can be caused by vacancy, substitution, or interstitial exchange of cations in the crystal structure. Hexagonal perovskites also tend to enable B‐site vacancies. There is a significant increase in the number of discovered B‐site deficient hexagonal perovskite oxides in recent years, establishing a solid foundation for a deeper expedition. Other than substitutional (A‐ or B‐site) stoichiometry compensation, some of the ions can also be located interstitially (A and/or B) that are called as interstitial defects.^[^ ^81^, ^82^ ^]^

#### 4.1.2 Anion or Oxygen Defects

**Figure 6:**
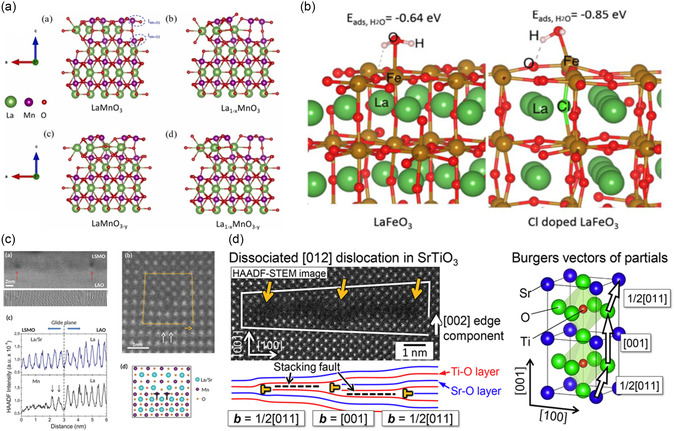
a) Side view of perovskite (001) surfaces: (a) LaMnO_3_, (b) La_1−*x*_MnO_3_, (c) LaMnO_3−*y*_, (d) La_1−*x*_MnO_3−*y*_ with cation deficiency. Reproduced with permission.^[^ ^126^ ^]^ Copyright 2024, Elsevier. b) Adsorption of H_2_O on anion deficient LaFeO_2.85_Cl_0.15_ perovskites. Reproduced with permission.^[^ ^127^ ^]^ Copyright 2019, Elsevier. c) Misfit dislocations in partially relaxed LSMO/LAO thin films. Reproduced with permission.^[^ ^128^ ^]^ Copyright 2018, Wiley. d) Magnified HAADF‐STEM images of two types of the dissociated dislocations with a [002] edge component. Reproduced with permission.^[^ ^129^ ^]^ Copyright 2017, Elsevier.

Oxygen vacancies are well‐known catalytically active sites. An increase in the oxygen vacancy concentration could result in anion defects. O‐site interstitial or oxygen interstitials can also be formed like A‐B interstitial sites. The vital role of anionic oxygen defects in improving electrocatalytic activity of perovskites has been extensively studied. For instance, Wang et al. confirmed that substitution of Ni in the Mn site of LSM perovskites resulted in the generation of oxygen vacancy and redox pairs Ni^3+^/Ni^2+^ and Mn^4+^/Mn^3+^, causing the surface atom rearrangement that facilitated the bifunctional activity.^[^ ^83^ ^]^ Introducing abundant oxygen vacancies in perovskites by partly replacing the cation has been recorded to improve oxygen catalysis, particularly OER. Similar findings have been recorded for other major catalytic reactions like ORR, HER, and electrochemical water splitting, as well. The side view of La‐based perovskite structures with cation and anion deficiencies created by density functional theory calculations is depicted in **Figure** **6**a,b for visualization.

As discussed earlier, an ideal perovskite exhibits a cubic Bravais lattice of space group Pm3m with a cubic crystal structure where central A ion is located within a cubic B sublattice, and each B cation is coordinated by six octahedral X ions. The ideal perovskite has a Goldschmidt tolerance factor (*t*) of 1. The formation of oxygen vacancies in perovskite oxides means there is a vacant space in an otherwise orderly crystalline structure. Oxygen vacancy thus leads to the expansion or tilt of the crystal lattice due to the vacancy which also influences the cation oxidation state. So, distorted perovskites with values outside of the range 0.7 > *t* > 1.0 are observed. The mismatched ionic radii could cause distortion in the BO_6_ octahedra that eventually results in the phase transformation to another polymorph. The main cause of these phase transformations are the oxygen vacancies formed by the A/B ratio. So far there are 23 possible variants of such tilts cited by Glazer and concised to 16 in accordance to symmetry.

### 4.2 Dimensional Defects

In 0D, the structural defects are mainly point defects.^[^ ^84^, ^85^ ^]^ There could be a cation or anion vacancy, and the corresponding charge substitution vacancies and vice versa without any charge compensation. The displacement of lattice ions (cations/anions) without charge substitution is known as an antisite defect. The formation of vacancy at A/B‐site and anion site simultaneously results in a Schottky pair. Instead of vacancy, if there is a displacement of cations or anions from their original site to an intersite or antisite, it results in a Frenkel defect or antisite defect, respectively. These defects are clearly illustrated in Figure 4. These point defects are majorly observed in thin film perovskite oxides or polycrystalline hybrid thin film perovskites.

1D structural defects are basically identified as lattice dislocations.^[^ ^86^ ^]^ There are two kinds of dislocations possible in the lattice structure. When there is a compression or tension created in the lattice due to insertion or desertion of the plane of ions, they are called edge dislocation. When there are shearing stresses not just in the plane but also the lattice, it is known as screw dislocation. The lattice dislocations and misfit observed from high‐angle annular dark‐field ‐ scanning transmission electron microscopy (HAADF‐STEM) can be observed in Figure 6c,d. 1D structural defects are prone to halide perovskite oxide nanowires applied for photovoltaic, photocatalysis, and laser applications.

2D structural defects are basically planar defects.^[^ ^87^ ^]^ They can be mainly classified into surface and interfacial defects. When these defects are found in the material interface of two phases or two grains in case of a bulk material, then it is called as phase boundary or grain boundary, respectively. There are also other defects like coherent boundary or incoherent boundary defects that are self‐explanatory. 2D materials are mostly layered structures, more specifically, RP, Dion–Jacobson, and Aurivillius perovskite oxides. The main defects in these layered structures can be stacking defects, which also plays a key role in their catalytic application. The stacking defect in RP oxides has been broadly utilized in energy storage and conversion application and catalysis. These defects are observed in both bulk and powdered nanomaterial crystalline perovskite oxides.

3D structural defects are volumetric defects.^[^ ^77^ ^]^ When there is a vacant space, it is commonly called as void. On the other hand, when there are multiple vacancies or ions, it is called clusters. However, depending on the size of the vacant space/occupant, the defects can vary from microporous (<2 nm), mesoporous (2–50 nm) to macroporous (>50 nm). Even fractured surfaces can constitute to structural defects. These defects can cause a great degree of disorder in the 3D structure of the perovskite oxides. This is mostly noticed in mesoporous perovskite oxides.

## 5 Defect Engineering of Perovskite Oxide Catalysts

Energy storage, conversion, and environmental remediation are the two main driving forces that propel catalysis research. The most prominent O‐related electrocatalysis processes OER and ORR (metal air battery), as well as OER and HER (electrochemical water splitting), are highly valued and have broad application prospects. Due to their structural and compositional flexibility, in addition to the ability to tailor and tune their characteristics, perovskite oxides are greatly investigated as low‐cost alternatives in electrocatalysis. An effective perovskite oxide catalyst is (expected to catalyze these reactions readily. To understand catalysis, it is necessary to understand the detailed reaction mechanism.

### 5.1 Oxygen Evolution Reaction Mechanism

**Figure 7:**
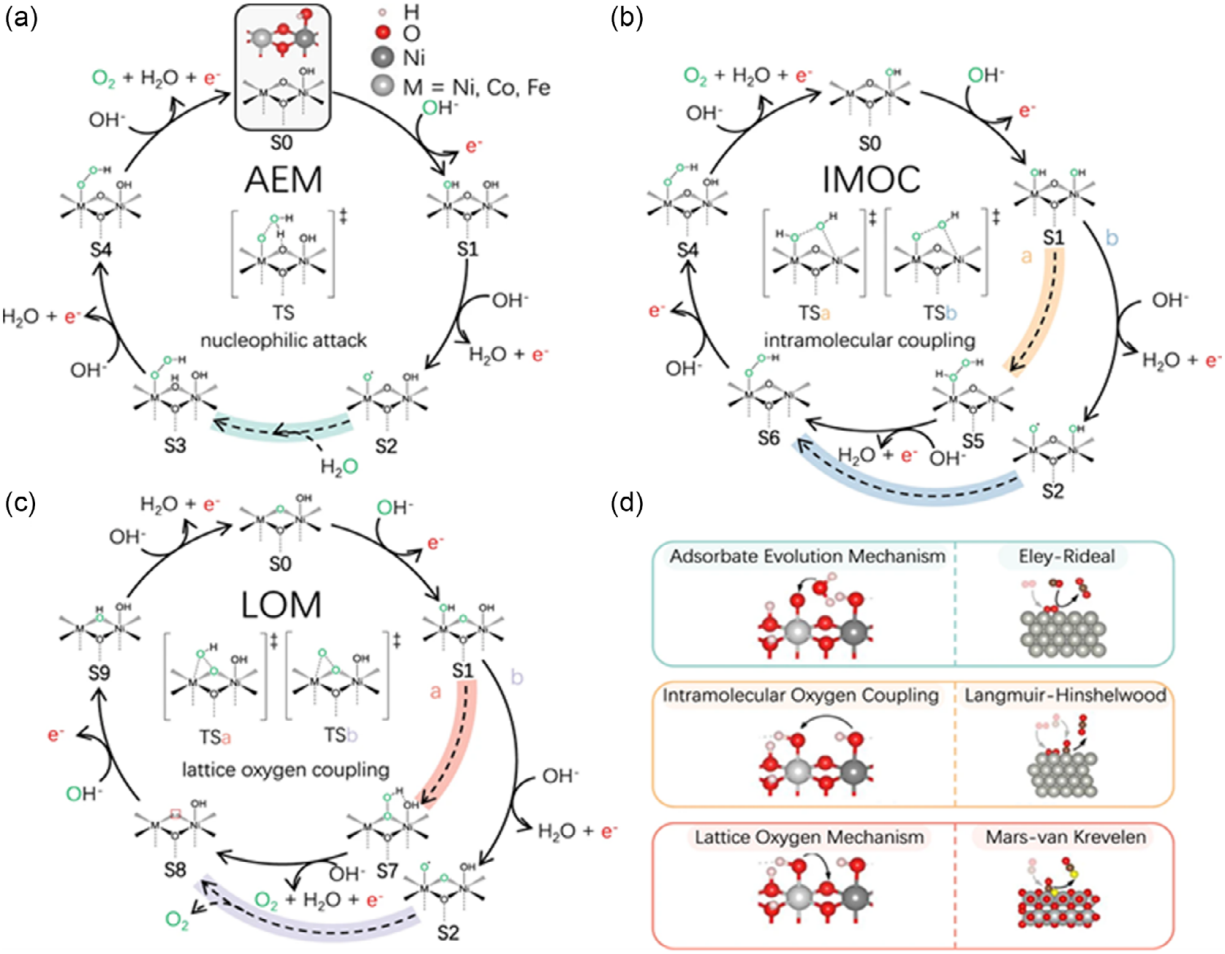
a–d) OER proposed mechanism in alkaline medium. Reproduced with permission.^[^ ^88^, ^130^ ^]^ Copyright 2021, American Chemical Society; 2023, Springer Nature.

The three possible proposed mechanisms of perovskite oxides for OER in both acidic and alkaline media are illustrated in **Figure** **7**a, namely, adsorbate mechanism (AEM), intramolecular oxygen coupling mechanism (IMOC), and lattice oxygen participation (LOM), of which AEM is the most commonly discussed.^[^ ^88^ ^]^ Generally, OER mechanism treads a four‐electron pathway that occurs on the active sites of the catalysts, particularly B‐site of the perovskite oxides. The first step of AEM is OH adsorption on the active site, i.e., the hydroxyl occupies the B‐site, resulting in the formation of M—OH. The adsorbate then dehydrogenates to surface‐adsorbed oxygen M—O. Due to the dehydrogenation, the M—O formation is usually the rate‐determining step of OER for most perovskite catalysts. The absorbed oxygen continues to react with the OH to form M—OOH and finally dehydrogenates the H, resulting in M—O=O where O_2_ is released. The steps are depicted in Figure 7a as (AEM) and S1–S4 denote the steps involved. When some perovskite oxides possess relatively high energy barriers, the reaction pathway alters to form a thermodynamically stable reaction pathway which is called as the LOM (Figure 7c). LOM starts with an oxygen vacancy in the lattice, following which the adjacent site of the catalyst adsorbs extra hydroxyl M_1_—OH/M_2_—OH. Then dehydrogenation generates M_1_—OH/M_2_—O, which is unstable and leads to a possible transition state (M—OHO) that further transits to M_1_—O—O/M_2_. The cycle completes by releasing O_2_. Although LOM mechanism can lower the energy barrier, only a few high covalent perovskites can undergo LOM. Other than AEM and LOM, catalysts also tend to follow the intramolecular O—O coupling mechanism. Here, the catalyst has high surface coverage and plenty of M–O formation, which struggles to form M—OOH. So, instead of the second step, a high energy barrier is required in the third step unlike AEM. Thus, M—O is easily coupled to adjacent M–O rather than forming M—OOH bonding, and generates O_2_, which gives the name for the mechanism (Figure 7b). Since IMOC mechanism requires conditions like a high energy barrier and the presence of two adsorbed oxygen adjacent to each other, this mechanism is rarely reported.^[^ ^89^ ^]^

### 5.2 Oxygen Reduction Reaction Mechanism

**Figure 8:**
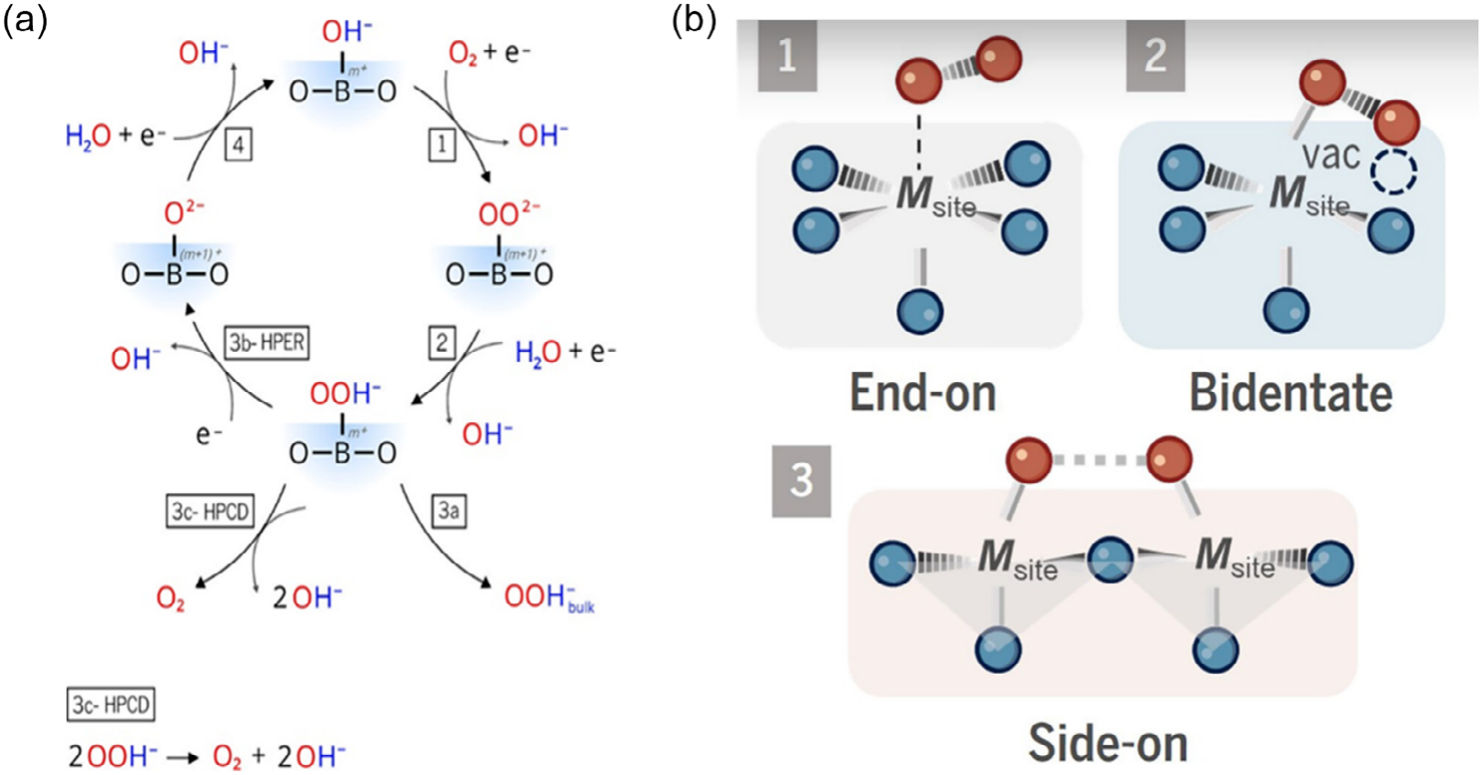
a) OER proposed mechanism in alkaline medium. Reproduced with permission.^[^ ^88^, ^130^ ^]^ Copyright 2023, Springer Nature. b) Three possible O_2_ adsorption configurations during ORR, including end‐on, bidentate, and side‐on. Reproduced with permission. Copyright 2024, Science.

The ORR mechanism for a perovskite single electrode in alkaline medium is shown in **Figure** **8**a. It should be noted that only specific catalysts with notably high free energy for oxygen adsorption (Pt) participate in acidic medium. ORR is the reverse reaction of OER. At the reducing potentials, the perovskite environment is enriched by OH ions that adsorbs onto perovskite surface. Now, O_2_ starts to adsorb onto the metal site of the active B‐site cations, replacing the adsorbed OH_ads_. This is the initial step which likely has three possibilities, namely, end‐on, bi‐dentate and side‐on, depending on the reverse reactions they follow, namely, AEM, LOM, and IMOC, respectively (Figure 8b). The as‐formed peroxide intermediates can desorb from the surface, react electrochemically in the hydrogen peroxide (HPER), or decompose chemically in the HPER chemical disproportionation reaction.^[^ ^90^ ^]^ The last step in the catalytic cycle is regeneration of the hydroxide surface (step 4). The ORR mechanism in perovskite oxides can be explained in two possible pathways, the two‐step, two‐electron pathway or the direct four‐electron transfer, of which the latter is more favorable and efficient. The 2‐by‐2 electron pathway includes
(2)

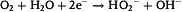



(3)

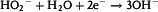

and the direct four‐electron reduction process:

(4) $$O_{2}+2H_{2}O+4e^{-}\to 4{\text{OH}}^{-}$$

It has been conclusively proven that highly defective perovskite oxides deliver better OER and ORR characteristics due to enhanced oxygen activation and conductivity.^[^ ^19^, ^25^, ^91^ ^]^ ORR is required to be performed in a rotating‐disc glassy carbon electrode, with rich supply of oxygen for a beneficial mass transport.

### 5.3 Hydrogen Evolution Reaction Mechanism

**Figure 9:**
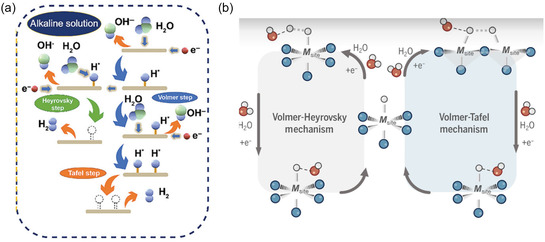
a) The schematic illustration of HER mechanism in alkaline media. Reproduced with permission. Copyright 2022, Wiley. b) Volmer–Heyrovsky and Volmer–Tafel mechanisms of HER. Reproduced with permission. Copyright 2024, Science Advances.

Hydrogen is considered as an ideal fuel for its abundance and zero‐emission or environmental benignity. One of the simplest and promising approaches to produce clean hydrogen is splitting water electrochemically by HER and OER reactions. HER is a two‐electron transfer reaction happening in the cathodic part of the water electrolyzer. Perovskites in general are involved in alkaline HER, as illustrated in **Figure** **9**a. Initially, the water dissociates and a proton is adsorbed onto the active B‐site to form H* in the Volmer step. Subsequently, H* readily combines with any other proton in the Heyrovsky step to release H_2_. However, H* could also combine with another H* where they both combine to release H_2_ in the Tafel step. Tafel slope derived from the Butler–Volmer equation is a common descriptor to determine the HER process, where the theoretical values correspond to 120, 40, and 30 mV dec^−1^ for Volmer, Heyrovsky, and Tafel steps, respectively. According to the experimental Tafel values, the HER can be called as Volmer–Tafel or Volmer–Heyrovsky mechanism (Figure 9b). Hybrid perovskite oxide structures are highly sought out for HER studies since SPs require a high activation energy for H adsorption, making it a rate‐limiting step. Hence, highly HER active metal oxides need to occupy the B‐site to achieve excellent HER performance.

### 5.4 Other Catalytic Reactions

Perovskite oxides also show promising catalytic properties in other catalytic reactions like nitrogen reduction reaction, carbon dioxide reduction reaction, urea oxidation reaction, and methanol oxidation reaction. The other details of these reactions are beyond the scope of this review.

### 5.5 A/B‐Site Cation Defects

Defect engineering is effectively pursued to optimize the electrocatalytic activity and bifunctionality of perovskite oxides. Ruo Qi Zong et al. used La_0.6_Sr_0.4_Co_0.2_Fe_0.8_O_3−*δ*_ (LSCF) perovskite to etch the A‐site element—Sr—by selective dissolution method, which increased the exposure of B‐site, decreased the coordination of B‐site metals, and favorably tuned the electronic structure of the material by introducing A‐site defect and oxygen vacancies.^[^ ^25^ ^]^ Gang Ou et al. applied lithium reduction method to engineer surface defect on LSCF perovskite as model and attempted to tune the electrocatalytic properties. According to the authors, lithium captured the lattice oxygen on the surface to form lithium oxide, thereby implanting defects at the surface of LSCF. In this case, the defects inserted a disordered layer of certain thickness, hence engineering the surface defective structure with enhanced bifunctional catalytic performance.^[^ ^92^ ^]^

**Figure 10:**
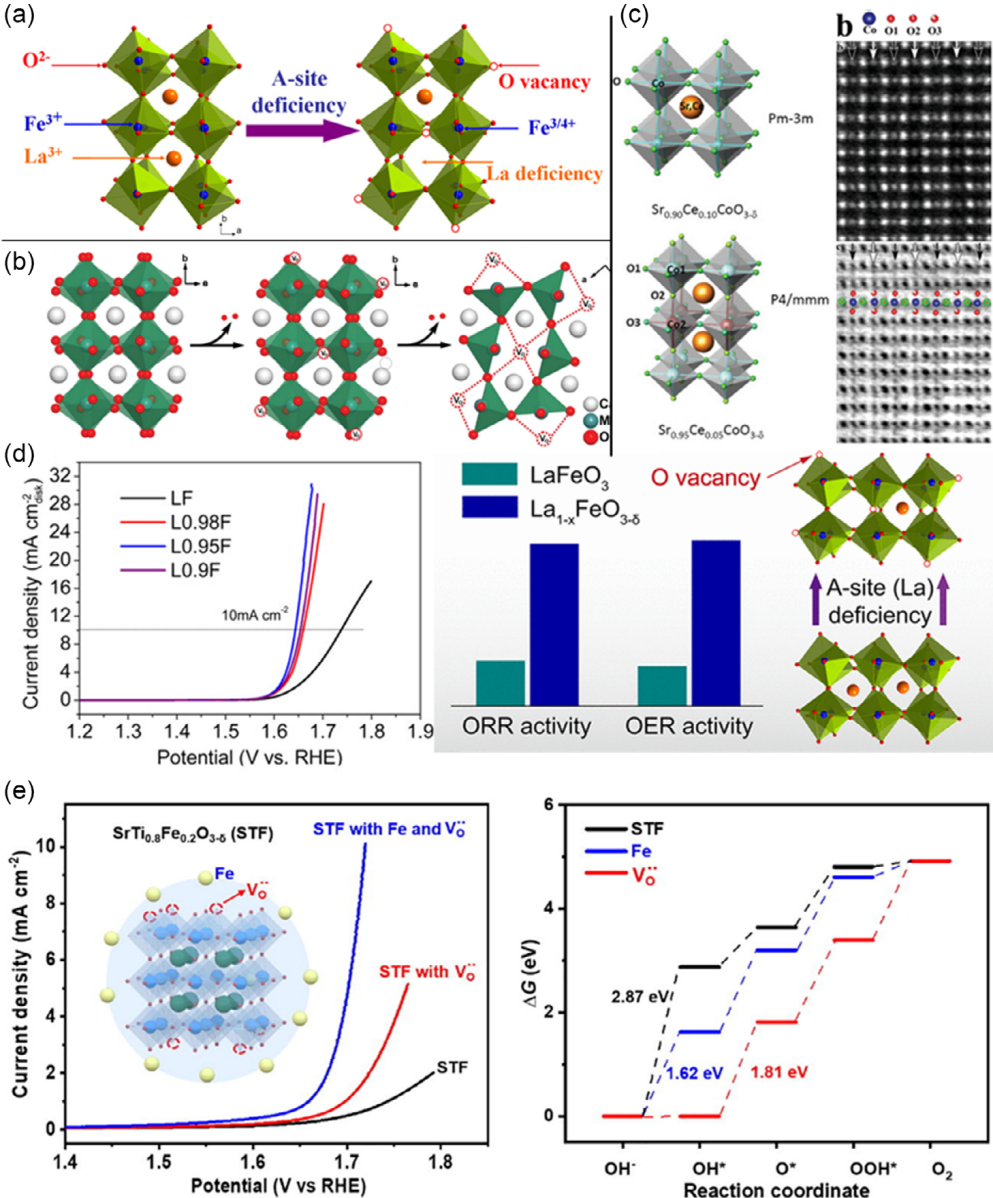
a) Schematic illustration of the formation of oxygen vacancy and Fe^4+^ in A‐site‐deficient La_1−*x*_FeO_3−*δ*_ perovskites and the corresponding electrocatalytic activity data is in d). Reproduced with permission.^[^ ^99^ ^]^ Copyright 2016, RSC. b) Schematic representation of the crystal frameworks of stoichiometric and oxygen‐deficient perovskites showing a transition from stoichiometric CaMnO_3_ to nonstoichiometric CaMnO_2.76_ and CaMnO_2.5_. Reproduced with permission.^[^ ^131^ ^]^ Copyright 2014, ACS. c) The corresponding crystal structures of Sr_0.90_Ce_0.10_CoO_3–*δ*_ and Sr_0.95_Ce_0.05_CoO_3–*δ*_.^[^ ^132^ ^]^ Right top: HAADF‐STEM image which shows A/B cation sites as well as high contrast in O_3_ site. Bottom: The ABF‐STEM image, from which the O‐deficiency at O_3_‐site was observed. Reproduced with permission.^[^ ^133^ ^]^ Copyright 2015, ACS. e) Enhanced OER activity achieved by surface‐decorated oxygen vacancies and Fe nanoparticles on STF and LTF perovskites, and the corresponding DFT calculation. Reproduced with permission.^[^ ^134^ ^]^ Copyright 2022, Elsevier.

Zhu et al. report an effective strategy for enhancing the bifunctional electrocatalytic activity of LaFeO_3_ simply by introducing A‐site cation, and La_0.95_FeO_3−*δ*_ (or L0.95F) delivered the best ORR and OER activity with high bifunctional index.^[^ ^90^ ^]^ The structural defects by introducing oxygen vacancies and cation deficiency are produced in **Figure** **10**a and the corresponding OER data obtained are included in Figure 10d. Du et al. examined the ORR and OER of a series of nonstoichiometric CaMnO_3−*δ*_ (0 < *δ* ≤ 0.5); the crystal structure modification by introducing oxygen vacancy is shown in Figure 10b.^[^ ^93^ ^]^ Experimental and theoretical calculations using density functional theory (DFT) confirm the activation of oxygen and improved conductivity, even exceeding the state‐of‐the‐art commercial catalysts like Pt and Ir, which results in the overall better performance of perovskite oxide structures. Sun and co‐workers examined perovskite Sr_1–*x*_Ce_*x*_CoO_3–*δ*_ (0.05 ≤ *x* ≤ 0.15) as cathodes for solid oxide fuel cells.^[^ ^94^ ^]^ When the structural modification of Sr_0.95_Ce_0.05_CoO_3–*δ*_ was studied by temperature‐dependent neutron powder diffraction, it revealed highly anisotropic displacement in the oxygen atoms of Sr_0.95_Ce_0.05_CoO_3–*δ*_ at 1100 K, indicating a large ionic mobility. Further scanning transmission electron microscopy (STEM) revealed the ordered oxygen vacancies in the structure (Figure 10c). This study concludes the dynamic breathing of B‐site octahedra, explaining the diffusion of oxygen ions in the structure by a vacancy mechanism. These examples concede that defective perovskite oxides deliver better performance due to the enhanced oxygen mobility and favorable structural refinement. Similarly, an enhanced OER activity achieved by surface‐decorated oxygen vacancies and Fe nanoparticles on SrTi_0.8_Fe_0.2_O_3−*δ*_ (STF) and La_0.66_Ti_0.8_Fe_0.2_O_3−*δ*_ (LTF) perovskites are shown in Figure 10e. Substitution or doping also effectively contributes in the defect engineering of perovskite oxides. Strontium is one of the most widely used A‐site dopants in perovskite oxides. Medford et al. methodically analyzed the OER activity of LSC with respect to Sr doping level.^[^ ^95^ ^]^ According to the author, the replacement of La^3+^ by Sr^2+^ led to higher oxygen‐deficiency‐enhanced covalency of the Co—O bond and eventually better OER activity. In detail, the OER activity was attested to the surface oxygen vacancy concentration. They also proposed that OER on LSC with Sr doping led to the exchange of lattice oxygen species, as confirmed by DFT and experimental results.

ABO*x* perovskite oxides like SrFeO_3_, CaFeO_3_, and BaFeO_3_ exhibit oxygen vacancy ordering in the form of channels.^[^ ^96^ ^]^ In some cases, ordered anionic deficiencies were found to be detrimental to OER catalysis. So cation substitution strategy was used to form ordered vacancies in BaFeO_3_. To reason this phenomenon, A‐ and B‐site substitutions mainly for disordering the oxygen vacancies were comparatively investigated. It was inferred that a donor dopant suppresses oxygen vacancy ordering and an acceptor dopant enhances the same. Another study shows a series of A‐site substituted (Pr_1−*x*_M_*x*_) MnO_3_ (M = Ca, Sr, Ba; *x* = 0–1.0) as electrocatalysts.^[^ ^97^ ^]^ The catalytic activities showed tremendous increase in the electron exchange in between Mn^3+^–Mn^4+^ pairs, Mn^3+^ being the active site cation. This study underscores that better electron exchange could be influenced by the difference in electronegativity of the ions. Xu et al. also recorded that the excess A‐site dopants could be charge‐compensated by B‐site vacancies.^[^ ^98^ ^]^ Alternatively, oxygen vacancies produced by A‐site doping could also be counterbalanced by the oxygen formation. In that case, A‐site excess would be balanced by B‐site valence.

Zhu et al. also reported improved bifunctional (ORR and OER) activity of La_1−*x*_FeO_3−*δ*_ (LF) perovskites in alkaline solution by creating A‐site cation deficiency (*x* = 0, 0.02, 0.05, 0.1).^[^ ^99^ ^]^ The surface oxygen vacancy concentration in the A‐site cation‐deficient La_1−*x*_FeO_3−*δ*_ perovskites along with the lesser Fe^4+^ species is the reason for the better ORR and OER activity. Moreover, Fe as the dopant has enriched the surface of the perovskite oxide that served as electrocatalytic active site. Similar transition metal ions at the surface could facilitate the adsorption and desorption of intermediates during the OER process. Very recently, Li et al. incorporated Ca into the LaMnO_3_ perovskite structure to create an exceptionally thin atomic layer named LCMO64.^[^ ^100^ ^]^ Author's observations revealed that leaching the A‐site Ca cation subsequently resulted in surface reconstruction and greatly increased the electrochemically active surface area with exceptional ORR activity.

**Figure 11:**
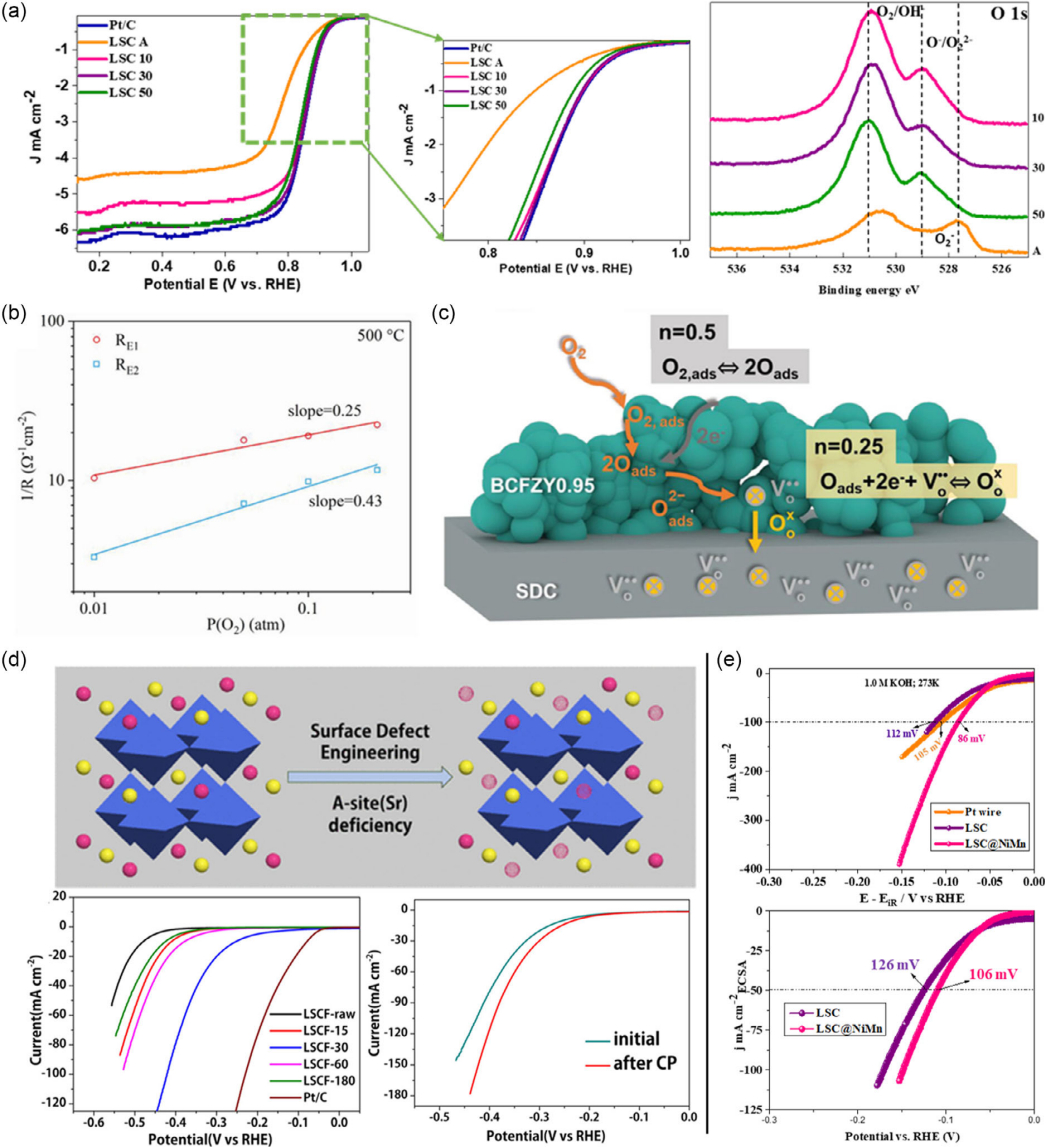
a) Enhanced ORR performance comparable to that of Pt/C by surface engineering and inducing oxygen vacancy in LSC. Reproduced with permission.^[^ ^11^ ^]^ Copy right 2019, ACS. b,c) Tafel slope and schematic diagram of oxygen reduction reaction over BCFZY0.95 electrodes with anion deficiency. Reproduced with permission.^[^ ^104^ ^]^ Copy right 2019, Wiley. d,e) Enhanced performance or HER with surface A‐site defect and interface engineering in La‐based catalysts. Reproduced with permission.^[^ ^25^, ^44^ ^]^ Copyright 2021, ACS; 2023, Elsevier.

B‐site is the most important active site for OER and ORR catalytic activity. According to Rao et al. the large A‐site cations at 12‐coordinated sites could be even partly missing since the BO_3_ in the perovskite structure forms a stable network.^[^ ^101^ ^]^ On the contrary, B‐site vacancies are firmly less favored because of the structural insecurity resulting in large architectural charge and the relatively smaller size of the B‐site cations. La (Ni_1−*x*_Fe_*x*_)O_3_ (*x* ≤ 0.6), with Ni and Fe B‐site, was reported by Zhang et al. as a bifunctional catalyst for metal–air batteries.^[^ ^102^ ^]^ The crystallinity of the material reportedly increased with the dopant level. According to the authors, Ni defects acted as nucleation sites and facilitated the recrystallization of Fe on the B‐site. It was observed that the surface was enriched in NiO, which could be detrimental to ORR and OER. Yet, Fe by recrystallization suppressed the formation of NiO and contributed to the catalytic activity, resulting in better OER and ORR. A similar study was performed with a series of B‐site metals, namely, LaMO_3_ (M = *d* block transition metals Cr, Mn, Fe, Co, and Ni).^[^ ^103^ ^]^ Depending on the B‐site cations, the catalytic properties varied interestingly. According to the authors, synergistic behavior enhances the electrocatalytic activity. For example, Mn^3+^ + Fe^3+^ à Mn^4+^ + Fe^2+^ and Ni^2+^ + Fe^3+^ à Ni^3+^ + Fe^2+^. So, by comparing the free energy, a synergistic combination for B‐site substitution can be selected. Kuai et al. introduced B‐site cation deficiency into BaCo_0.4_Fe_0.4_Zr_0.1_Y_0.1_O_3−*δ*_ (BCFZY) perovskite lattice structure and the resulting Ba(Co_0.4_Fe_0.4_Zr_0.1_Y_0.1_)_0.975_O_3−*δ*_ (BCFZY0.975) and Ba(Co_0.4_Fe_0.4_Zr_0.1_Y_0.1_)_0.95_O_3−*δ*_ (BCFZY0.95) perovskite oxides showed exceptional activity for ORR.^[^ ^104^ ^]^ The report concluded that by introducing B‐site cation deficiency the free lattice volume increased, resulting in the facilitation of oxygen diffusion within the oxide lattice and improved ORR performance. The enhanced ORR performance of LSC perovskite by enhanced oxygen vacancy is shown in **Figure** **11**a, where the O 1*s* X‐ray photon spectroscopy spectra show the oxygen vacancy enrichment by surface engineering the catalyst.

Apart from OER and ORR activities, defect engineering is a promising strategy for optimizing electrocatalytic HER. As discussed in HER mechanism, for HER, the H_ads_ must be adjacent to complete the Tafel mechanism and produce H_2_. However, it is not as simple in perovskite oxides which commonly exhibits Heyrovsky mechanism for HER. Zhao et al, compared the perovskite oxides with their respective B‐site metals and analyzed the Δ*G*_H_ values. It is noteworthy that Δ*G*_H_ close to 0 eV shows high HER activity and favorable adsorption and desorption of H. Hence, the overall activity mainly depends on the Δ*G*_H_ value of the elements occupying the B‐site metals. A‐site electronegativity prediction of (Gd_0.5_La_0.5_)BaCo_2_O_5.5+*δ*_ exceeded the state‐of‐the‐art Pt/C catalyst in HER activity and stability. Consecutively, the A‐site defective La‐based electrocatalyst exhibits exceptional HER after surface defect engineering of A‐site, as depicted in Figure 11d. Further, interface engineering of B‐site La‐based catalysts exhibits improved performance by anchoring chemical bonds and altering the crystalline and electronic structure of the catalyst (Figure 11e).^[^ ^44^ ^]^ Perovskite performance is predictable from accessible A‐site properties like ionic radii. Apart from that, a crucial electronic characteristic of A‐sites is the ion electronegativity which involves the structural and thermodynamic factors, which is a unifying parameter that also reflect B‐site characteristics.

### 5.6 A–B‐Site Defects

Ahmed et al. examined the redox and catalytic characteristics of the La_1−*x*_Sr_*x*_Fe_0.50_Co_0.50_O_3_ nanocatalysts with A/B‐site substitution.^[^ ^105^ ^]^ The X‐ray diffraction analysis of the Co‐substituted shows an orthorhombic structure with space group Pnma II and rhombohedral structure with space group R‐3c. With the further increase, there was more modification into cubic structure with space group Pm‐3m. Maria et al. have synthesized a DP (A_2_BB′O_6_) with different cations in A, B, and B′ sites, namely, (La_1.5_Sr_0.5_)_A_(Ni_0.5_Mn_0.5_)_B_(Ni_0.5_Ru_0.5_)_B′_O_6_ (LSNMR), with an outstanding OER/ORR bifunctional performance.^[^ ^106^ ^]^ DFT calculations revealed that the high bifunctional activity of LSNMR is related to the presence of active Mn sites for the ORR‐ and Ru‐active sites for the OER by virtue of the symmetric respective reaction steps. Pr (Ba_1−*x*_Sr_*x*_) (Co_1−*x*_Fe_*x*_) O_5+d_ with A/B substitution was synthesized and the oxygen concentration was varied postsynthesis.^[^ ^107^ ^]^ Although A‐site doping appeared irrelevant, Sr was expected to cause structural distortion. According to the authors, through the difference in electronegativity Sr facilitated the amorphization and promoted the disorder in the structure which eventually resulted in the enhanced OER activity. Simultaneous substitution of A‐ and B‐sites definitely shows an increase in oxygen vacancy which also influences the electrocatalytic activities like OER and ORR positively. A similar observation is reported for OER catalysis where using A, B‐site cations and oxygen vacancies has resulted in the exceptional performance for Bi_0.15_Sr_0.85_Co_1−*x*_Fe_*x*_O_3−*δ*_ (*x* = 0.2, 0.4, 0.6, 0.8, 1) perovskites in alkaline media. In this case, B‐site cation and oxygen vacancy have together played an effective role in the OER performances via surface reconstruction.^[^ ^81^ ^]^

### 5.7 X‐Site or Anion Defects

Anion defects can also critically impact the specific properties of the materials like electronic structure, ionic/electronic conductivities, and so on. Anion defects that commonly exist in metal oxide perovskites, namely, surface substitutional impurity atom, single oxygen surface vacancy, single oxygen bulk vacancy, substitutional impurity atom, interstitial impurity atom, and line defect, are shown in Figure 4. Kim et al. prepared single‐phase oxygen‐deficient perovskite Ca_2_Mn_2_O_5_ with intrinsic molecular‐level porosity on the oxygen‐deficient sites, which was expected to favor the ion transport during OER.^[^ ^108^ ^]^ As expected, the oxygen‐deficient Ca_2_Mn_2_O_5_ delivered an enhanced OER performance. According to Kim et al. the unit cell structure of the oxygen‐deficient Ca_2_Mn_2_O_5_ facilitated the facile transport of OH^−^ ions and also modified the electronic configuration of B‐site cations with a high‐spin electron occupying *e*_g_ orbitals, all through the oxygen vacancies (refer to Figure 10). Furthermore, Lyu et al. introduced oxygen deficiencies into CaMnO_3_ by substituting Nb into the Mn site of CaMnO_3_ and concluded that oxygen deficiency contributes to numerous factors, including phase stability, optimal *e*_g_ filling level, OH^−^ adsorption ability, and improved conductivity.^[^ ^109^ ^]^ Du et al. also observed similar enhancement in the ORR and OER activity of CaMnO_3_ due to oxygen defects. In all these studies, the oxygen defects were introduced by thermal treatment.^[^ ^93^ ^]^ Chen et al. reported the phase transformation of reconstruction Pr_0.5_Ba_0.5_MnO_3−*δ*_ (PBM) into layered PrBaMn_2_O_5+*δ*_ (H‐PBM) by thermal treatment which created oxygen defects and showed great activity for ORR and OER activity.^[^ ^110^ ^]^ Thermal treatment assists in the generation of oxygen vacancies and in certain cases like above, they results in the exsolution of secondary active phases that can significantly enhance the activity of the material. Sometimes, the phase separation and their interactions can also create additional active sites for favorable electrocatalytic activity. Moreover, the effect of anion doping has a direct effect on the oxygen vacancy concentration. When the ions substitute the lattice oxygen directly, they maintain the electric neutrality in the system by decreasing the oxidation state of neighboring metal cations and losing the lattice oxygen, which will result in an increase in the oxygen vacancy concentration. Surface oxygen exchange activity can rapidly enhance the performance of perovskite materials depending on the rapid oxygen exchange at the reaction interface, which can drive the catalytic reaction kinetics favorably. According to the theory by Suntivich et al. the active redox pairs located in the highest energy level of O 2*p* band of the B—O molecular orbital model depend on the covalency between B‐site and oxygen.^[^ ^111^ ^]^ Thus, the stronger covalency between the B‐site element and oxygen will result in higher OER performance. However, for a deeper understanding, the integration of DFT calculations with various novel in situ characterization techniques is essential. A summary of recent defective perovskite oxides for bifunctional electrocatalysis is provided in **Table** **1**.

**Table 1:** Summary of recent defective perovskite oxides for bifunctional electrocatalysis.

Perovskite catalyst	Defect	Method	Application	Medium	References
LCMO64	Doping	A‐site cation	ORR	0.1 m KOH	2024^[^ ^77^ ^]^
La_0.6_Sr_0.4_CoO_3−*δ*_	Oxygen vacancy	In situ chemical bonding	OER	1 m KOH	2023^[^ ^40^ ^]^
La–Sr–Co–Fe–O	Oxygen vacancy	A‐site cation deficiency	OER	0.1 m KOH	2022^[^ ^82^ ^]^
La_0.9_Sn_0.1_NiO_3−*δ*_	Oxygen vacancy and orbital occupancy	A‐site cation substitutions	OER	0.1 m KOH	2022^[^ ^83^ ^]^
Sr_2_Co_1.5_Fe_0.5_O_6−*δ*_	Oxygen vacancy	Disorderly orientating	OER	0.1 m KOH	2021^[^ ^84^ ^]^
LaMnO_3_	Oxygen deficiency	Treatment	ORR/OER	0.1 m KOH	2021^[^ ^85^ ^]^
5.84% S–LaMO_3_ (M = Co, Fe, Ni)	B‐site substitution	Elemental doping	ORR/OER	0.1 m KOH	2020^[^ ^86^ ^]^
Sr_2_Fe_1.5_Mo_0.5_O_6*x*_dF_*x*_	Occupancy	Elemental doping	OER	0.1 m KOH	2020^[^ ^87^ ^]^
Ba_0.9_Sr_0.1_Co_0.8_Fe_0.1_Ir_0.1_O_3−*δ*_	Occupancy	Elemental doping	OER	0.1 m KOH	2020^[^ ^130^ ^]^
LaCoO_3_	Oxygen vacancy and orbital occupancy	A‐site deficiency	ORR/OER	0.1 m KOH	2020^[^ ^88^ ^]^
SrRuO_3_, Sr_0.95_Na_0.05_RuO_3_, and Sr_0.90_Na_0.10_RuO_3_	Vacancy	Elemental doping	OER	0.1 m HClO_4_	2019^[^ ^89^ ^]^
(La_0.8_Sr_0.2_)_1+*x*_MnO_3_	Orbital occupancy	B‐site deficiency	ORR/OER	0.1 m KOH	2019^[^ ^76^ ^]^
NdNiO_3_	Oxygen vacancy and orbital occupancy	Elemental doping	ORR/OER	0.1 m KOH	2019^[^ ^90^ ^]^
La_0.8_Sr_0.2_MnO_3_	Oxygen vacancy	Elemental doping	ORR/OER	0.1 m KOH	2019^[^ ^91^ ^]^
La_0.8_Sr_0.2_MnO_3_ (LSM)	Oxygen vacancy	Elemental doping	ORR/OER	0.1 m KOH	2018^[^ ^92^ ^]^
CaMnO_3_	Oxygen vacancy	Elemental doping	ORR/OER	0.1 m KOH	2018^[^ ^93^ ^]^

**Figure 12:**
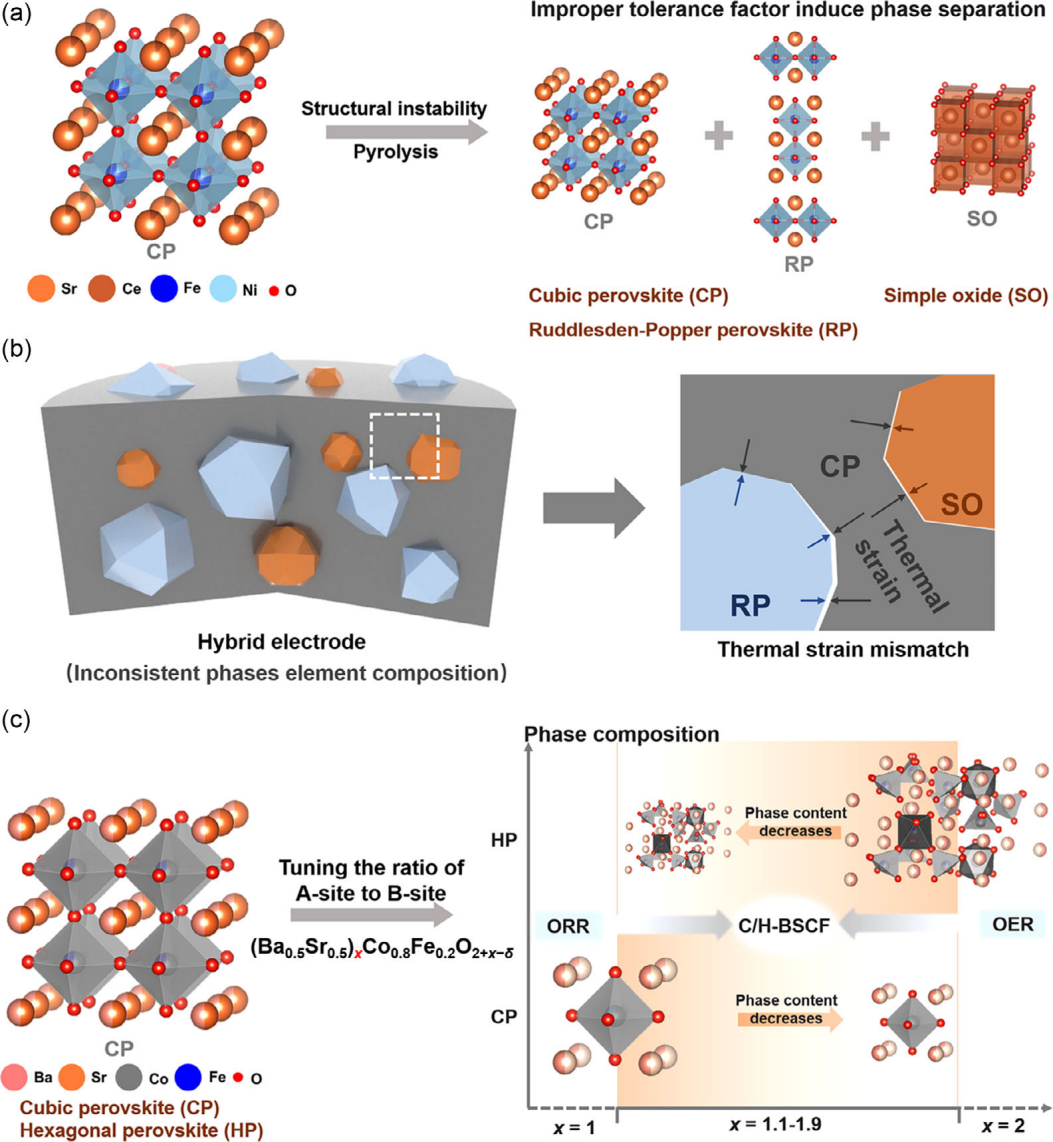
a) The hybrid electrodes formed by structural instability and pyrolysis induce phase separation due to an improper tolerance factor of the partial cation in the bulk phase, thus forming a RP perovskite or simple oxide. b) Mismatched thermal stress between multiple phases of hybrid electrodes synthesized by improper tolerance factor. c) The phase content‐controlled hybrid electrode composed of cubic and hexagonal perovskites induced by tuning the ratio of A‐site to B‐site of cubic perovskite. Reproduced with permission.^[^ ^114^ ^]^ Copyright 2024, Nature.

The formation of oxygen vacancies in perovskite oxides means there is a vacant space in an otherwise orderly crystalline structure. Oxygen vacancy thus leads to the expansion or tilt of the crystal lattice due to the vacancy which also influences the cation oxidation state resulting in phase transformation. Polymorph perovskite oxide catalyst has been reported to contribute superfluous active sites with high conductivity. They are also said to exhibit synergy with the structural transformation and multiphase. Song et al. reported Sr_0.9_Ce_0.1_Fe_0.8_Ni_0.2_O_3−*δ*_ (SCFN) surface‐enriched with CeO_2_ and NiO nanoparticles where the combination of tetragonal and RP structures delivers rapid oxygen kinetics using the multiphase synergy.^[^ ^112^ ^]^ Similarly, Liang et al. utilized cation tailoring of cubic Ba_0.95_(Co_0.4_Fe_0.4_Zr_0.1_Y_0.1_)_0.95_Ni_0.05_O_3−*δ*_ (BCFZYN) with NiO and realized excellent proton conduction.^[^ ^113^ ^]^ It has been recorded that with a sturdy coupling of a SP structure and RP layered phase, extraordinary oxygen activation can be achieved for OER. Very recently, Liu et al. prepared a dual‐phase hybrid perovskite oxide using A‐site modulation of the classic perovskite oxide BSCF (**Figure** **12**).^[^ ^114^ ^]^ According to the report, the DFT calculations reveal that the cubic and hexagonal perovskites during ORR and OER reactions exhibit synergistic effect of both the phases resulting in an beneficial accord.

### 5.8 Other Defects

There are innumerable methods to alter the stoichiometry of the perovskite oxide catalysts using defects. One of the widely used idea is by controlled extrinsic doping by donors or acceptors in A or B or A–B‐sites. Nevertheless, in recent days, intrinsic and extrinsic doping (by donors or acceptors) have been reported to achieve the purpose of defect engineering, resulting in an improved physicochemical characteristic of the material. Further, in some cases, mixed donor and acceptor ions at the A or B or A–B‐sites could induce a charge compensation resulting in the generation of oxygen vacancies.

#### 5.8.1 Jahn–Teller Distortion

Jahn–Teller distortion is a concept that when a molecule is degenerate at the ground state, the lattice will distort in order to adapt to a more stable energy level. Very recently, Whittingham et al. by tuning the composition of layered perovskite oxides and systematically analyzing the local distortions, have reported the fundamental aspects of electrochemical reaction pathways.^[^ ^115^ ^]^ The authors have compared the effect of composition‐dependent structural parameters on the ability of La_1.2_Sr_0.8_Ni_1–*y*_Co_*y*_O_4_ to catalyze ORR. The study concludes that the Co content and the magnitude of anisotropic lattice compression are relatively proportional which is attributed to the Jahn–Teller expansion of Ni/Co—O_ax_ bonds. That is, the magnitude of the distortion altered the measure of catalytic activity, proving the significance of the effect on catalytic performance. This study assures that Jahn–Teller distortion can also be included as an effective defect engineering strategy.

To summarize, in optimizing perovskite oxides for OER, one of the simplest way is by substituting A‐site metal with stable La‐based perovskite elements like Ba and Sr. This ensures the structural stability of the OER catalyst. Second, B‐site element can be replaced with the combination of multimetallic *d* block elements such as NiFe or NiMnFeCu to enhance the electronic as well as other intrinsic properties. In addition, O‐site modification will have a direct effect on the formation and performance of perovskite oxides. For designing perovskite oxides for ORR, the choice of B‐site metals is the priority which must possess high activity. B‐site metal is responsible for *e*_g_ filling and for optimizing the oxygen vacancy concentration. *e*_g_ orbital filling can be controlled by substitution at A/B‐sites or by modulating the distribution of electron in the BO_6_ octahedron. Oxygen vacancies can also be regulated by substituting A‐sites or nonprecious metals. For finding the best suitable perovskite oxides for HER, it is important to understand that perovskite oxides are not ideal catalysts for HER. So to achieve efficient perovskite oxides for HER, it is crucial to mind the Δ*G*_H_ value of the occupying metal center. It is even simpler to choose the best suitable metal with appropriate Δ*G*_H_ value for the B‐site of the perovskite oxides. Recent findings have established that Ru‐based perovskite oxides like SrRuO_3_ with high activity, are excellent choice for HER performance. Considering this, high‐activity Ru‐based perovskite catalysts can be prepared with minimal utilization of Ru with varying parameters and interfacial engineering. Despite the catalytic reactions, and regardless of OER, ORR, and HER there are few techniques that are always favorable for catalysis. One technique is by sintering the perovskite oxides at high temperature which rejuvenates the structure and properties of the materials. Another is to use different gaseous atmospheres in order to mobilize and enhance the active lattice oxygen and oxygen vacancies.

## 6 Summary and Future Perspective

Perovskite materials are highly interesting materials sought out by researchers of versatile fields owing to their structure, flexibility, tunability, and intrinsic properties. Defect engineering embodies a precise approach toward the improvement of OER and ORR performance of perovskite oxide catalysts. Though many have achieved successful improvement of catalytic activity through other descriptors, there are still many challenges remaining to be overcome in defect engineering. To deduce a plausible conclusion, it is also necessary to combine experimental conclusions with modern theoretical calculations like DFT. It can predict the transformation in the local electronic structure induced by defects and draw a reliable reaction pathway for the reactions. This review unveils the relationship between crystal structure and catalytic performance of perovskite structures. Future research in defective perovskites needs to prioritize the following: 1) Optimizing the synthesis process: crystal formation and composition are vital to understand the defects in perovskite crystallization. In situ monitoring could help in the formation of desired phases and understand the nucleation mechanism. 2) Atomic‐scale analysis: characterizations like transmission electron microscopy in atomic resolution can provide insights into detailed atomic structure and precisely show the localization of defect sites. Hence, atomic‐scale analysis is crucial for defect regulation in perovskite oxide catalysts. 3) Simulation and calculation: first‐principle DFT calculations and high‐resolution scanning tunneling microscopy can be used to understand the chemical identity and nature of defects. Highly stable surface structured perovskites can be successfully characterized if the nature of defects can be analyzed.

## Conflict of Interest

The authors declare no conflict of interest.

## Author Contributions

**Maria Christy**: Conceptualization (supporting). **Seunggun Choi**: Conceptualization (supporting). **Jiseok Kwon**: Conceptualization (supporting). **Jinwoo Jeong**: Project administration (supporting); Supervision (supporting). **Ungyu Paik**: Conceptualization (supporting). **Taeseup Song**: Conceptualization (supporting).
